# Polyethylene Terephthalate Glycol-Modified (PETG) as a Reusable and Biocompatible Substrate for Cell Culture Applications

**DOI:** 10.3390/jfb17070336

**Published:** 2026-07-11

**Authors:** Alessia Vita, Federica Tiberio, Diego Sibilia, Martina Salvati, Domiziano Dario Tosi, Lorena Di Pietro, Antonio Alliva, Carlo Mariella, Ornella Parolini, Wanda Lattanzi

**Affiliations:** 1 Dipartimento Scienze Della Vita e Sanità Pubblica, Università Cattolica del Sacro Cuore, Largo F. Vito 1, 00168 Rome, Italy; alessia.vita@unicatt.it (A.V.); federica.tiberio@unicatt.it (F.T.); martina.salvati@unicatt.it (M.S.); lorena.dipietro@unicatt.it (L.D.P.); ornella.parolini@unicatt.it (O.P.); 2 Fondazione Policlinico Universitario A. Gemelli, IRCCS, Largo A. Gemelli 8, 00168 Rome, Italy; diego.sibilia1@unicatt.it; 3 Unità Operativa Complessa di Neurochirurgia Infantile, Fondazione Policlinico Universitario A. Gemelli, IRCCS, Largo A. Gemelli 8, 00168 Rome, Italy; domizianodario.tosi@unicatt.it; 4 3DiTALY Roma, Circonvallazione Casilina 137/139, 00176 Rome, Italy; antonio.alliva@3ditaly.it (A.A.); carlo.mariella@3ditaly.it (C.M.); 5 Fondazione IRCCS Casa Sollievo Della Sofferenza, Viale Cappuccini, 71013 San Giovanni Rotondo, Italy

**Keywords:** polyethylene terephthalate glycol, green transition, circular economy, cell culture substrates, research laboratory plastic

## Abstract

The development of reusable and biocompatible biomaterial-based culture substrates is increasingly relevant for improving sustainability in biomedical research workflows. In this study, polyethylene terephthalate glycol-modified (PETG) was evaluated as a potential alternative to conventional polystyrene (PS) for in vitro cell culture applications. PETG substrates were fabricated through laser cutting and tested for their ability to support cell adhesion, viability, proliferation, and lineage-specific differentiation across multiple human cell models, including calvarial mesenchymal stromal cells (CMSCs), bone marrow-derived mesenchymal stromal cells (hBM-MSCs), dermal fibroblasts, LHCN-M2 myoblasts, and SH-SY5Y neuroblastoma cells. Morphological and immunofluorescence analyses demonstrated that PETG supported cell attachment and focal adhesion formation, comparable to standard PS surfaces. Cell viability and proliferation assays confirmed metabolic activity and growth over time. Furthermore, PETG substrates supported osteogenic, adipogenic, myogenic, and neuronal differentiation, as demonstrated by histological staining, myotube formation, neurite outgrowth, and lineage-specific gene expression analyses. Finally, PETG maintained CMSC morphology and metabolic activity after repeated recovery, ethanol/UV treatment, and gelatin re-coating, with comparable results between new substrates and those reused for up to three cycles. These findings support PETG as a biocompatible culture substrate with preliminary short-term reuse potential and possible sustainability benefits for laboratory workflows.

## 1. Introduction

Plastic pollution is one of the most critical environmental challenges of our time, driven by the rapid and ongoing increase in plastic production and use. An estimated 360 million tons of plastic materials are produced each year, with a turnover of over 350 billion euros for the European plastics industry [1,2,3]. Due to their favorable thermal and mechanical properties, plastics are employed in a wide range of applications across various sectors, including agriculture, industry, automotive, sports and medicine. Single-use applications, such as packaging, represent the majority of plastic use [4]. However, these same properties that make plastics so valuable also contribute to their environmental persistence in ecosystems as macro- and microplastics, posing significant threats to biodiversity and human health.

In recent years, significant attention has been directed toward mitigating plastic pollution through strategies such as recycling, reduction in single-use plastics, and the adoption of circular economy principles. Despite these efforts, the rate of recycling remains unsatisfactory to offset the environmental burden, with only a fraction of plastic waste being effectively recovered or repurposed [5]. This imbalance underscores the need for innovative material design and waste management strategies to align with global sustainability goals.

Beyond industrial and consumer applications, biomedical and research laboratories substantially contribute to plastic waste generation because of their strong reliance on disposable consumables required to maintain sterility, biosafety, and experimental reproducibility. Common laboratory items, including pipette tips, tubes, and cell culture vessels, are primarily manufactured from petroleum-derived polymers such as polystyrene (PS), polypropylene (PP), polyethylene (PE), and polymethyl methacrylate (PMMA), and are routinely discarded after a single use [6]. Consequently, laboratory activities generate considerable quantities of plastic waste, while current recycling systems remain poorly adapted to manage biologically contaminated materials [7,8,9]. These limitations highlight the need for reusable and recyclable materials compatible with routine biomedical workflows and circular economy strategies.

Several bio-based and biodegradable polyesters have recently been investigated as more sustainable polymeric platforms for biomedical and tissue engineering applications [10,11]. Among these, polylactic acid (PLA), polycaprolactone (PCL), polyhydroxyalkanoates (PHAs), polybutylene succinate (PBS), and related biodegradable blends or copolymers have received considerable attention because of their processability, biodegradability, and potential bio-based origin [12,13]. These materials have been widely explored for scaffold fabrication, regenerative medicine, drug delivery, and tissue engineering applications [11]. However, their translation into routine laboratory consumables and cell culture workflows may be limited by material-dependent constraints, including sterilization sensitivity, degradation-related changes in surface and mechanical properties, and variability in long-term stability under culture conditions [10,11].

As an alternative to biodegradable materials, durable and recyclable thermoplastics may offer a complementary strategy to reduce reliance on single-use laboratory plastics. Polyethylene terephthalate (PET) is a thermoplastic polyester with high recyclability, bioinertness, chemical resistance, and mechanical durability, making it potentially suitable for circular economy-oriented applications [9,14]. Building on its widespread use in packaging and textiles, PET benefits from established recycling pathways, further supporting its potential for sustainable applications.

Among PET-derived materials, polyethylene terephthalate glycol-modified (PETG) has gained attention as a versatile thermoplastic polyester [15]. PETG is an amorphous polymer obtained through glycol modification of PET, which enhances flexibility, transparency, impact strength, chemical resistance, and processability [16]. Although PETG is not resistant to standard autoclaving, it can be processed by thermoforming, laser cutting, and additive manufacturing, and has been reported for food and water contact applications [17,18].

In biomedical contexts, PETG has been employed for producing vascular and orthopedic prostheses, mammography phantoms, and custom-made surgical implants, owing to its biocompatibility and ease of processing [19,20,21,22,23]. Notably, while PETG has been widely used for mold fabrication in cranial implant procedures, studies evaluating its direct in vivo use remain limited, highlighting the need for further exploration of its biomedical potential [24,25,26].

More recently, PETG has also been investigated in additive manufacturing and biomedical device fabrication, including the optimization of 3D-printing parameters for cranial implant design [27], PETG-based composite scaffolds for bone tissue repair [28], and evaluation of cell adhesion on 3D-printed PETG surfaces [29]. These studies are consistent with recent reviews highlighting the growing relevance of 3D-printed polymeric scaffolds and customized biomaterial platforms in biomedical applications [30,31]. Overall, the available evidence supports the biomedical interest in PETG, particularly for customized three-dimensional structures, scaffolds, and device-oriented applications.

However, most available PETG-based biomaterial studies focus on 3D-printed constructs, scaffolds, or patient-specific devices, whereas less attention has been given to simple flat PETG sheet substrates designed for routine two-dimensional cell culture workflows. Therefore, the novelty of the present study does not lie in the general use of PETG as a biomedical material, but in the evaluation of laser-cut, optically transparent, flat PETG sheet substrates as practical culture supports compatible with standard culture plates and microscopy-based workflows. This approach differs from previously reported PETG-based biomaterial studies mainly focused on three-dimensional architectures or device fabrication, and combines a simple substrate format with a multi-cell-type biological evaluation and preliminary short-term reuse assessment.

The aim of this study was to evaluate laser-cut PETG sheet substrates as alternative supports for routine two-dimensional cell culture applications. PETG substrates were assessed for their ability to support cell adhesion, viability, proliferation, and differentiation across different adherent cell types. In addition, we evaluated their compatibility with common cell biology workflows, including live-cell analysis, microscopy-based assays, immunofluorescence, RNA extraction, and gene expression analysis. Finally, a short-term repeated reuse experiment was performed to preliminarily assess whether PETG substrates could maintain cell compatibility after recovery, ethanol/UV sterilization, and re-coating cycles. By combining flat laser-cut substrate fabrication with a multi-cell-type biological testing and preliminary reuse assessment, this study explores PETG as a practical and potentially reusable substrate for cell culture applications.

## 2. Materials and Methods

### 2.1. Production of PETG Substrates

PETG substrates for cell culture were produced using a subtractive manufacturing process based on laser cutting (BEAMBOX PRO, Flux Inc., Taipei, Taiwan) from commercially available transparent extruded PETG sheets (Mayku Clear Sheet 0.5 mm, Dongguan Wan Su Cheng Plastic Co., Ltd., Dongguan, China). The manufacturer-reported composition and technical properties of the virgin PETG sheet selected for substrate fabrication are summarized in Table 1. The substrates were designed using 2D CAD (Computer-Aided Design) software (AutoCAD 2024; Autodesk, San Rafael, CA, USA) to ensure compatibility with standard 6-well culture plates. The design was then processed by CNC (Computer Numerical Control)-guided laser cutting to obtain PETG disks with the desired geometry and dimensions. Laser parameters, including power, cutting speed, and pulse frequency, were optimized to ensure reproducible cutting and dimensional consistency. The production process was carried out in collaboration with 3DiTALY (https://www.3ditaly.it/roma/; accessed on 10 July 2026).

### 2.2. PETG Sterilization and Surface Coating

Before cell seeding, PETG substrates underwent sequential low-temperature sterilization and gelatin coating. Initially, the substrates were immersed in an 80% ethanol solution (Sigma-Aldrich, Darmstadt, Germany) for 30 min, followed by two rinses with PBS (Dulbecco’s PBS without calcium and magnesium, Aurogene, Rome, Italy) to remove ethanol residues. Sterilization was completed by UV exposure under a laminar flow hood. Since PETG is not resistant to standard autoclaving conditions, ethanol/UV treatment was selected as a practical sterilization approach compatible with the thermal properties of the material.

Following sterilization, PETG substrates were coated with gelatin to improve cell adhesion. Surface coating is a common practice for laboratory culture supports (i.e., multi-well plates and flasks) to enhance cell adhesion and growth [32]. Among various coatings, gelatin, a denatured form of collagen, was selected for our substrates owing to its cost-effectiveness and ability to support cell attachment while preserving a natural morphology [32]. Before selecting the final coating condition, preliminary qualitative observations were performed using CMSCs cultured for 24 h under proliferative conditions on uncoated PETG substrates and PETG substrates coated with 0.1% gelatin. Cells were able to adhere under both conditions, indicating that PETG was compatible with cell attachment. However, gelatin-coated PETG showed a more homogeneous cell distribution and a more reproducible cell morphology across the substrate surface (Supplementary Figure S1). Preliminary observations indicated that poly-L-lysine, although effective in promoting adhesion, induced morphological changes in some cell types; therefore, this coating was not selected for the subsequent experiments.

For gelatin coating, PETG substrates were treated with a 0.1% gelatin solution (0.1% in water; STEMCELL Technologies™, Vancouver, BC, Canada) at room temperature for 20 min to promote cell adhesion. Excess solution was removed, and the substrates were air-dried before being transferred into 6-well plates for subsequent cell seeding in culture experiments. Accordingly, the cellular responses reported in this study should be interpreted as responses to the PETG–gelatin culture interface rather than to pristine PETG alone.

### 2.3. Scanning Electron Microscopy Analysis

Surface morphology of conventional polystyrene (PS), laser-cut PETG sheet substrates, and fused deposition modeling-produced PETG substrates (PETG-FDM) was evaluated by scanning electron microscopy (SEM). Representative substrate specimens from each material were mounted on aluminum supports using double-sided carbon tape. Before imaging, samples were sputter-coated with a 10 nm layer of platinum to reduce charging artifacts. SEM images were acquired using a Supra 25 microscope (Zeiss, Oberkochen, Germany) in In-Lens secondary electron mode, at an accelerating voltage of 10 kV, scan speed 4, line averaging (*n* = 3) for noise reduction, and a working distance of 1 mm. Images were collected at 200×, 500×, and 1000× magnification to qualitatively compare surface morphology, continuity, porosity, and manufacturing-related topographical features among PS, laser-cut PETG, and PETG-FDM substrates.

### 2.4. Cell Culture on PETG

To evaluate the feasibility of PETG as a cell culture substrate, both primary cells and cell lines were employed. All cell types were seeded using a “drop-based” sedimentation method to promote efficient cell attachment. Specifically, a 100 µL droplet, containing the required cell density, was placed at the center of each PETG substrate. After 4–6 h of incubation, 1.5 mL of culture medium was gently added to each well to ensure optimal growth conditions. The specific culture conditions for each cell type employed are detailed in the following sections.

#### 2.4.1. Calvarial Mesenchymal Stromal Cell Culture

Calvarial mesenchymal stromal cells (CMSCs) were selected as osteoprogenitors to evaluate the biocompatibility of PETG substrates and their osteogenic potential. CMSCs were previously isolated in primary culture from cranial bone tissue collected as surgical waste from craniosynostosis patients (Ethical Committee-approved protocol-Prot. N. 0001276/24), according to standardized protocols [33,34]. To evaluate cell adhesion, cell-biomaterial interactions, viability, and cell proliferation, CMSCs were seeded at a density of 5 × 10^3^ cells/cm^2^ on PETG and PS supports and cultured with a proliferative medium consisting of Dulbecco’s Modified Eagle Medium high-glucose (DMEM HG; Aurogene) supplemented with 1% L-glutamine (Euroclone, Milan, Italy), 1% antibiotics (penicillin 100 U/mL, streptomycin 100 µg/mL; Euroclone), and 10% Fetal Bovine Serum (FBS; Aurogene) at 37 °C with 5% CO_2_ for different time points.

In addition, to study the osteogenic differentiation of CMSCs, cells were seeded on PETG and PS substrates, as described above, and cultured for 72 h (T0) in the proliferative medium. Subsequently, the medium was replaced with an osteoinductive medium (OM) containing dexamethasone (0.1 µM), β-glycerol phosphate (0.1 µM), ascorbic acid (50 nM) (Sigma-Aldrich, Saint Louis, MO, USA), 10% FBS (GIBCO, ThermoFisher Scientific, Waltham, MA, USA), 1% antibiotics (penicillin 100 IU/mL, streptomycin 100 µg/mL), 1% L-glutamine, and DMEM low-glucose (1 g/L; Aurogene). The OM was changed every 2 days up to 7 days (7 d).

#### 2.4.2. Human Bone Marrow-Derived Mesenchymal Stromal Cell Culture

Primary human bone marrow-derived mesenchymal stromal cells (hBM-MSCs) were selected for their multilineage differentiation potential [35] to assess the applicability of PETG-based supports, including their ability to sustain adipogenic differentiation.

hBM-MSCs were purchased from STEMCELL Technologies (Cat. No. 70001) and seeded at a density of 5 × 10^3^ cells/cm^2^ on PETG and PS supports to investigate the capacity of PETG to promote cell adhesion and viability at different time points. hBM-MSCs on both substrates were cultured in a growth medium (GM; MesenCult MSC Basal Medium (STEMCELL Technologies)) supplemented with Mesencult MSC Stimulatory Supplement (STEMCELL Technologies). To further investigate the effect of PETG on cell differentiation processes, hBM-MSCs were induced to undergo adipogenic differentiation for 10 days. At 72 h post-seeding, confluent cells on both substrates were incubated with an adipogenic induction medium (AM1) containing DMEM HG with 10% FBS GIBCO, 0.5 mM 3-isobutyl-1-methylxanthine (IBMX; Sigma-Aldrich, St. Louis, MO, USA), 0.25 µM dexamethasone (Sigma-Aldrich), 10 µg/mL insulin (Sigma-Aldrich), 1% L-glutamine, and 1% antibiotics. After 72 h in AM1, cells were switched to an adipogenic maintenance medium (AM2) containing DMEM HG, 10% FBS GIBCO, 10 µg/mL insulin, 1% L-glutamine, and 1% antibiotics for 7 days.

#### 2.4.3. Human Dermal Fibroblasts Culture

Human dermal fibroblasts, purchased from Cytion (Human Dermal Fibroblasts—Adult (HDF-Ad), Cytion, Eppelheim, Germany; Cat. No. 300606), were selected owing to their central role in extracellular matrix synthesis and tissue remodeling [36], making them an ideal model for assessing how biomaterials influence cell growth and function. Cells were seeded on PETG and PS supports at a density of 5 × 10^3^ cells/cm^2^ and cultured with DMEM HG medium supplemented with 20% FBS, 1% L-glutamine and 1% antibiotics at 37 °C with 5% CO_2_. Cell adhesion, viability, and proliferation on PETG were evaluated and compared to PS at different time points, as described below (see Section 3.2.1, Section 3.2.2, Section 3.2.3 and Section 3.2.4).

#### 2.4.4. LHCN-M2 Human Myoblast Cell Line Culture

The LHCN-M2 human myoblast cell line (Evercyte, Vienna, Austria) was selected to evaluate myogenic potential. Cells were initially expanded at a density of 1.2 × 10^3^ cells/cm^2^ in T75 flasks pre-coated with 0.1% gelatin using Myoup complete medium (Evercyte) to support cell proliferation without inducing differentiation. Myoblasts were subsequently seeded on 6-well PETG and PS plates at a density of 2 × 10^4^ cells/well in DMEM HG with 10% FBS to assess cell adhesion, viability, and proliferation as well as cell-biomaterial interactions, compared to standard laboratory plastics, at different time points. To evaluate the potential of PETG to sustain the myogenic differentiation of LHCN-M2 myoblasts into myotubes, cells were cultured in an induction medium consisting of four parts DMEM HG mixed with one part Medium 199 (Gibco, Thermo Fisher Scientific, Waltham, MA, USA), supplemented with 2% horse serum (HS, Gibco, Thermo Fisher Scientific).

#### 2.4.5. SH-SY5Y Neuroblastoma Cell Line Culture

The SH-SY5Y neuroblastoma cell line (ATCC^®^ CRL-2266™, Manassas, VA, USA) was chosen for its relevance in neuronal studies and its capability for differentiation into neuron-like cells. SH-SY5Y cells were plated at 3 × 10^4^ cells/well on PETG and PS supports and cultured with DMEM HG supplemented with 10% FBS, 1% antibiotics, 1% L-glutamine and 1% MEM non-essential amino acids (Thermo Fisher Scientific), at 37 °C with 5% CO_2_. The medium was changed twice weekly for different time points to determine cell adhesion and viability. In addition, neuronal differentiation was induced by incubating SH-SY5Y with DMEM HG supplemented with 1% FBS, 1% antibiotics, and 10 μM all-trans-retinoic acid (RA, Cat. A6947, >98% pure, Panreac AppliChem ITW Reagents, Barcelona, Spain) (differentiation medium #1) for 7 days. RA promotes the differentiation of SH-SY5Y cells from a neuroblastic to a neuron-like morphology, reducing proliferation and enhancing neuron-like characteristics. After 7 days, the medium was removed and replaced by differentiation medium #2 consisting of DMEM HG, 1% FBS, 1% antibiotics, and 2% B27 supplement (Thermo Fisher Scientific) for 3 days to support neuronal health and maturation.

### 2.5. Morphological Evaluations

The initial stage of the evaluation of PETG’s biocompatibility entailed a morphological analysis to compare cell adhesion and shape on PETG versus PS substrates across all cell types. This analysis was conducted to identify any observable alterations in cell morphology that might suggest differences in cell–substrate interactions or cell health between the two materials. Cell morphology was assessed using a Primovert Zeiss microscope (Carl Zeiss Inc., Thornwood, NY, USA), with magnifications of 4×, 10×, and 20×, to capture both general cell distribution and detailed cellular characteristics. Each analysis was conducted in biological triplicates, with 12 images captured per replicate at different magnifications.

### 2.6. Cell Adhesion After Washing Assay

To quantitatively assess CMSCs adhesion after washing, CMSCs were seeded on PS and gelatin-coated PETG substrates at a density of 5 × 10^3^ cells/cm^2^ using the drop-based sedimentation method described in Section 2.4. At 6, 12, and 24 h after seeding, substrates were gently washed with sterile PBS to remove non-adherent or loosely attached cells. The remaining adherent cells were then detached and counted using acridine orange/propidium iodide staining, performed with the CellDrop AO/PI Viability Assay kit (Cat. No. CD-AO/PI-1.5; DeNovix Inc., Wilmington, DE, USA). This assay discriminates viable and non-viable nucleated cells based on acridine orange and propidium iodide fluorescence. Cell adhesion after washing was expressed as the percentage of viable adherent cells recovered after washing relative to the initial number of seeded cells. Each condition was performed in biological triplicates.

### 2.7. Immunochemistry Analysis

To evaluate cellular interactions with PETG, immunofluorescence staining for vinculin was performed in CMSCs and myoblasts cultured on PETG and compared with cells seeded on PS. Briefly, both cell types were cultured on PETG and PS under proliferative conditions, as previously described. After 48 h, cells were first washed with PBS, fixed with 4% (*v*/*v*) formaldehyde solution, and permeabilized using 0.5% Triton X-100 (Sigma-Aldrich) in PBS. Then, cells were incubated with 1% BSA (Sigma-Aldrich) in PBS at 37 °C for 5 min to block non-specific binding sites. Blocking was followed by incubation with an anti-vinculin primary antibody (1:100, #V9131, Sigma-Aldrich) for 1 h at 37 °C. After washing, cells were incubated with an Alexa Fluor 488-conjugated secondary antibody (1:200, #715-545-150, Jackson ImmunoResearch, West Grove, PA, USA) for 1 h. Myoblasts were also incubated with an anti-β-actin primary antibody (1:200, #8457, Cell Signaling Danvers, MA, USA) for 1 h at 37 °C. After washing, a Cy3-conjugated secondary antibody (1:200, #705-165-147, Jackson ImmunoResearch, West Grove, PA, USA) was applied for 1 h to visualize β-actin in red. Nuclei were labeled with DAPI (5 µg/mL; Sigma-Aldrich). Fluorescence microscopy (Zeiss AXIO Imager, Jena, Germany) was used to capture images of focal adhesions and nuclei in CMSCs and myoblasts. Images were obtained using the Zen 3.1 software (Blue Edition, Carl Zeiss Micro Imaging, Jena, Germany). To quantify vinculin levels in CMSCs, a total of 12 non-overlapping fields were acquired for each condition. Vinculin fluorescence intensity was quantified using ImageJ software (Version 2.1.0/1.53c). The images were analyzed by applying a threshold adjustment, and the mean gray value was then calculated using the software’s default algorithm [37].

Fluorescence intensity quantification in myoblasts was performed using ImageJ by first separating the merged images into individual channels corresponding to vinculin (green) and β-actin (red). The intensity analysis was then carried out by applying threshold adjustments to enhance signal specificity, followed by the measurement of mean fluorescence intensity using the “Analyze > Measure” function on a total of 12 images per condition. All analyses were performed in biological triplicates for reproducibility.

### 2.8. Cell Viability Assay

Viability of CMSCs, hBM-MSCs, fibroblasts, myoblasts, and SH-SY5Y cultured on PETG supports was assessed by the CellTiter-Glo^®^ assay (Luminescent Cell Viability Assay; Promega, Madison, WI, USA), which quantifies adenosine triphosphate (ATP) levels as an indicator of metabolically active cells. Briefly, after 48 h of cell culture, the CellTiter-Glo^®^ reagent was added directly to the culture medium. Samples were shaken for 2 min to ensure complete lysis, then equilibrated at room temperature for 30 min. Luminescence intensity was recorded with Cytation 3 cell imaging multi-mode reader (Biotek, Winooski, VT, USA). The luminescent signal, proportional to ATP levels, provided a measure of cell viability for each substrate condition. Each condition was performed in biological triplicates.

### 2.9. Proliferation Analysis

Cell proliferation was tracked over 72 h (every 24 h) using the IncuCyte^®^ live-cell analysis system (Sartorius, Germany), an automated imaging platform that captures time-lapse images in a 37 °C incubator. By comparing the proliferation rates of cells on PETG and PS substrates, the IncuCyte^®^ provided detailed quantitative data on cell growth dynamics. Proliferation analysis was specifically conducted on CMSC, fibroblast, and myoblast cultures in their respective growth media. Each condition was conducted in biological triplicates to ensure data reliability. Cell proliferation was further characterized by gene expression analyses of stemness and proliferation markers in CMSCs cultured on PETG compared to PS (for further details see Section 2.13).

### 2.10. Alizarin Red Staining

To phenotypically assess the osteogenesis of CMSCs on both substrates, cells were fixed with 4% formalin and then stained with Alizarin Red S (ARS; Sigma-Aldrich) which binds to calcium ions in the mineralized matrix deposited by osteoblasts. Stained bone matrix was quantified by incubating the stained cultures with a 10% acetic acid solution for 30 min at room temperature to extract ARS. Following this, ammonium hydroxide (0.1 M) was added to neutralize the acid, and the absorbance of the extracted solution was measured at 405 nm using the Cytation 3 reader. This method provided an accurate measure of the mineralization extent and osteogenic differentiation of the cells. The osteogenic assay of cells on PETG was also evaluated by gene expression analysis of osteogenic markers (for further details see Section 2.13).

### 2.11. Oil Red O Staining

To determine the efficiency of adipogenic differentiation, hBM-MSCs were stained with Oil Red O (ORO; Sigma-Aldrich), a dye that specifically binds to neutral lipids within mature adipocytes. Briefly, cells were fixed in 10% formalin, washed with 60% isopropanol, stained with ORO for 10 min, then rinsed using distilled water to remove excess dye. For quantification of stained lipid droplets, 9 independent fields for each condition were captured using a Primovert Zeiss microscope. To quantify the intracellular lipid accumulation, ORO-stained lipid droplets were eluted with 100% isopropanol for 10 min. The optical density was measured at 520 nm using the Cytation 3 reader. The adipogenic differentiation of cells on PETG was also evaluated by adipogenic marker expression analysis (for further details see Section 2.13).

### 2.12. Immunofluorescence Staining for MHC

Myogenic differentiation of LHCN-M2 myoblasts was assessed using immunofluorescence to detect morphological changes. Cell samples were rehydrated in PBS, permeabilized with cold methanol at − 20 °C for 6 min and washed with PBS on a rocking platform. A blocking solution of 5% normal donkey serum (Sigma-Aldrich) in PBS was applied before incubation with the anti-MHC primary antibody (MF20, Hybridoma Bank, Iowa City, IA, USA) at 1:20 in PBS with 1% BSA for 1 h at room temperature, with gentle agitation. Following incubation, excess antibody was removed through PBS washes, and cells were incubated with Alexa Fluor 488-conjugated secondary antibody (1:100 #715-545-150, Jackson ImmunoResearch, West Grove, PA, USA). Nuclei were labeled with DAPI at concentration of 5 µg/mL for 5 min at 30 °C. Images were captured using a ZEISS Axio Imager fluorescence microscope, acquiring 12 independent fields per sample at 10× magnification with ZEN (3.1) Blue edition software. ImageJ software was used to quantify the total number of nuclei and determine the fusion index, representing the extent of myogenic differentiation. Additionally, gene expression analysis was performed to assess the expression of key genes involved in muscle differentiation pathways (see Section 2.13).

### 2.13. RNA Isolation and Gene Expression Analysis

After 72 h of proliferation and at the end of each specific differentiation time point, total RNA was extracted using TRIzol Reagent (Zymo Research Corporation, Irvine, CA, USA) according to the manufacturer’s protocol. RNA was quantified through a droplet/microvolume spectrophotometer NanoDrop (NanoDrop OneC—UV-Vis Spectrophotometer, Thermo Fisher Scientific) and 5 ng of RNA was reverse transcribed into cDNA using the PrimeScript™ RT reagent Kit (TaKaRa Bio USA, Inc., Mountain View, CA, USA). The quantification of expression levels of specific stemness, proliferation and differentiation markers (Table 2) was assessed by real-time PCR (qPCR) using the Syber Green system with GoTaq^®^ qPCR Master Mix (Promega, Madison, WI, USA). Real-time PCR was carried out through a QuantStudio 1™ Real-Time PCR system (Applied Biosystems, Foster City, CA, USA). Each sample was analyzed at least in duplicate.

The expression levels of all target genes were normalized to the β-actin housekeeping gene and calculated using the 2^−ΔΔCt^ method [38]. Primer sequences used for amplification are listed in Table 2.

### 2.14. CMSCs Culture on Single-Use PETG and Reused PETG Substrates

#### 2.14.1. Preliminary Single-Reuse Assessment

To assess the potential of PETG for repeated use in cell culture applications, previously utilized PETG substrates were sterilized and reused without undergoing re-extrusion or additional material processing. The goal was to determine whether reused PETG maintained the structural and functional properties necessary for effective cell culture. Briefly, the substrates were initially washed twice with PBS to remove loosely attached cellular debris, minimizing surface contamination. Once the absence of visible cellular material was confirmed using a microscope, the substrates were immersed in 80% ethanol for 30 min under laminar flow. Sterilization was completed by exposing the supports to UV radiation for 1 h, with 30 min of exposure on each side, targeting microbial contaminants on surface-exposed areas. Following sterilization, the reused PETG substrates were tested for their ability to support cell growth. To this aim, CMSCs were seeded at a density of 5 × 10^3^ cells/cm^2^ on reused PETG using the previously described “drop-based” sedimentation method and cultured in a proliferative medium (DMEM HG) for 72 h to qualitatively assess cell attachment, spreading, morphology, and apparent cell growth. CMSC adhesion and morphology were qualitatively evaluated by light microscopy at 6, 24, and 72 h after seeding. Cells seeded on single-use PETG were used as controls.

#### 2.14.2. Cell Viability Assessment After Repeated PETG Reuse Cycles

To assess whether short-term repeated reuse affected the ability of PETG substrates to support CMSC viability/metabolic activity, PETG substrates were evaluated after consecutive 48 h culture/recovery/sterilization/re-coating cycles. Four conditions were analyzed: non-reused PETG substrates, used as controls, and PETG substrates reused once, twice, or three times, hereafter referred to as PETG-new, PETG-R1, PETG-R2, and PETG-R3, respectively. For each 48h use cycle, CMSCs were seeded on PETG substrates at a density of 5 × 10^3^ cells/cm^2^ using the drop-based sedimentation method described in Section 2.4 and cultured in proliferative medium. At the end of each 48h culture period, representative light microscopy images were acquired to document CMSC adhesion and morphology. Cell viability/metabolic activity was then assessed using the CellTiter-Glo^®^ assay, as described in Section 2.8, and luminescence values were reported as absolute relative luminescence units.

Since the CellTiter-Glo^®^ is an endpoint destructive assay, each reuse stage included independent replicate PETG substrates dedicated either to CellTiter-Glo^®^ analysis or to continuation to the following reuse cycle. Therefore, substrates used for CellTiter-Glo^®^ measurements were not reused for subsequent cycles. Substrates intended for continuation were washed twice with sterile PBS, visually inspected to confirm the absence of visible residual cellular debris, sterilized using the ethanol/UV protocol described in Section 2.2, and re-coated with 0.1% gelatin before the next use cycle. Thus, PETG-new, PETG-R1, PETG-R2, and PETG-R3 were compared after the same 48 h CMSC culture duration, differing only in the number of previous reuse cycles.

### 2.15. Statistical Analysis

Data were analyzed using GraphPad Prism software version 11.0 (San Diego, CA, USA). Results are presented as the mean ± standard deviation (SD), with data obtained from biological triplicates or quadruplicates (*n* = 3–4), as indicated in the corresponding figure legends. Statistical differences between two groups were determined using an unpaired Student’s *t*-test. For experiments involving more than two groups, statistical analysis was performed using either one-way or two-way analysis of variance (ANOVA), depending on the number of independent variables, followed by Tukey’s multiple comparison test, as the appropriate post hoc analysis.

## 3. Results and Discussion

### 3.1. Design, Production, and Characteristics of PETG Substrates

**Figure 1:**
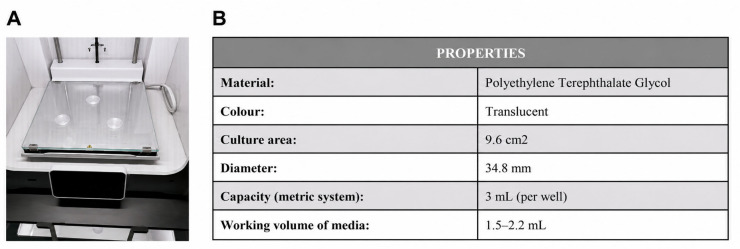
Production and properties of polyethylene terephthalate glycol-modified (PETG) substrates. (**A**) Representative image of PETG substrates production through subtractive manufacturing; (**B**) The table shows physical properties of PETG substrates.

PETG substrates were fabricated using a subtractive manufacturing process known as laser cutting, applied to extruded, optically transparent PETG panels (Figure 1A). This high-precision method enabled the production of substrates with defined geometric specifications, ensuring compatibility with the dimensions of standard cell culture plastic supports, particularly those of 6-well culture plates. The PETG substrates exhibited an optically transparent appearance, allowing clear observation of cell cultures under both optical and fluorescence microscopy. Each substrate offered a culture area of 9.6 cm^2^, providing an adequate surface for cell attachment and growth. The supports had a diameter of 34.8 mm, with each well accommodating a total volume of 3 mL and a working medium volume ranging from 1.5 to 2.2 mL, aligning with standard culture plate specifications (Figure 1B). These dimensions were carefully optimized to balance sufficient medium volumes for cell culture with minimal waste while maximizing the surface area for cellular interactions. Overall, the material properties and design of the PETG substrates made them ideal for cell culture applications, providing a reliable surface for cell adhesion and growth, while also allowing for efficient observation and handling of cultures. The detailed characteristics of the PETG supports are summarized in Figure 1.

Several studies have investigated the application of advanced 3D printing techniques, such as fused deposition modeling (FDM), for the fabrication of biomaterials tailored for tissue engineering. These include 3D PETG scaffolds with pore structures and enhanced mechanical properties, promising for bone tissue engineering applications, where controlled porosity supports osteogenic differentiation [20]. Although an FDM-produced PETG (PETG-FDM) substrate is a strong candidate for scaffold fabrication, our previous experimental data suggested that its properties were less suitable for flat substrates intended for direct cell culture, as required in standard in vitro models (Supplementary Figure S2). Specifically, the layered deposition process resulted in highly irregular surfaces, which compromised cell adhesion and visualization (Supplementary Figure S2). We observed that cells frequently became trapped within microvoids and interlayer spaces, leading to heterogeneous cell adhesion, altered growth patterns, and reduced viability, correlating with poor surface homogeneity and increased roughness (Supplementary Figure S2). Additionally, printed PETG supports exhibited increased opacity, requiring advanced imaging techniques such as confocal or electron microscopy, which are not compatible with routine laboratory workflows. Given these findings, our data indicated that FDM-based fabrication was not the optimal method for producing PETG-based cell culture substrates owing to inherent process limitations (see Supplementary Figure S2 for details). For this reason, high-precision subtractive manufacturing was then employed to produce uniform, optically transparent PETG substrates with a more regular surface morphology, ensuring optimal cell adhesion, viability, and reproducibility. This method eliminates layering artifacts and porosity issues associated with FDM, resulting in smoother and more consistent surfaces, thereby facilitating standard microscopic analysis and improving experimental reproducibility.

**Figure 2:**
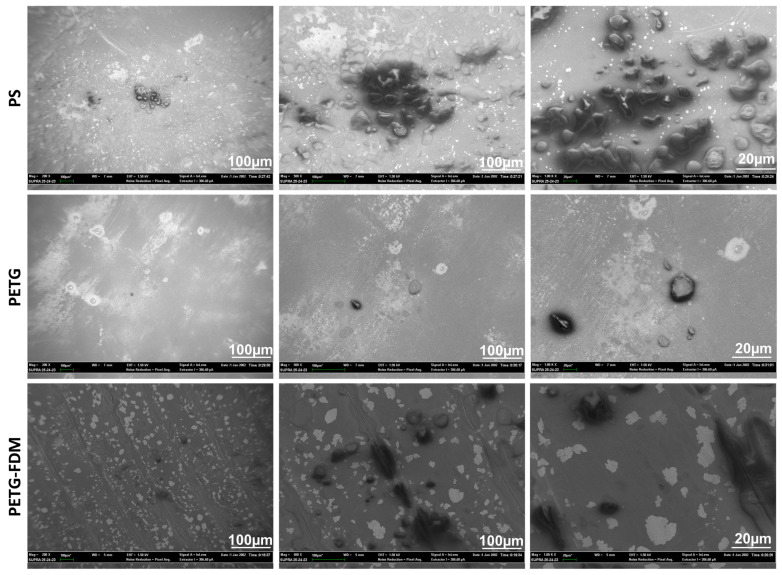
SEM surface characterization of PS, laser-cut PETG, and PETG-FDM substrates. Representative scanning electron microscopy images show the surface morphology of conventional polystyrene (PS), laser-cut polyethylene terephthalate glycol-modified (PETG) sheet substrates, and fused deposition modeling-produced PETG substrates (PETG-FDM). Images were acquired at 200×, 500×, and 1000× magnification. Scale bars are shown in each image. Scale bars: 100–20 µm.

To further support the suitability of laser-cut PETG as a flat cell culture substrate, SEM analysis was performed to compare the surface morphology of conventional PS, laser-cut PETG, and PETG-FDM substrates at 200×, 500×, and 1000× magnification (Figure 2). Conventional PS and laser-cut PETG displayed comparable compact and non-porous surface morphologies, without evident large pores, cracks, or major structural discontinuities at the analyzed magnifications. This indicates that the laser-cut PETG sheet provides a continuous culture surface morphologically similar to standard PS, supporting its use as an alternative flat substrate for routine two-dimensional cell culture. Minor superficial irregularities and occasional particulate or deposit-like features were observed on both PS and PETG surfaces, likely related to sample handling, preparation, or imaging artifacts. In contrast, PETG-FDM substrates exhibited a markedly more heterogeneous morphology, characterized by visible ridges, grooves, depressions, and pore-like interfilament discontinuities, consistent with the layer-by-layer deposition process typical of FDM fabrication. These SEM observations indicate that laser-cut PETG provides a compact, continuous, and non-porous culture surface that is morphologically comparable to conventional PS, with no evident large pores, cracks, or major structural discontinuities at the analyzed magnifications. By contrast, PETG-FDM displayed a less homogeneous and markedly more irregular surface architecture. Overall, these findings support the use of laser-cut PETG sheets as PS-comparable flat substrates for routine two-dimensional cell culture applications, while further justifying the exclusion of FDM-produced PETG from the main biological assays.

### 3.2. Biocompatibility Study on PETG

Biocompatibility is a critical parameter in selecting materials for cell culture applications that reflects the material’s ability to support cellular functions without causing adverse effects. A variety of cell types were employed to assess the versatility and compatibility of PETG substrates. These included human mesenchymal stromal cells (MSCs) from different sources (CMSCs; BM-MSCs), a human dermal fibroblast cell line, the LHCN-M2 human myoblast cell line and the SH-SY5Y human neuroblastoma cell line. Each cell type was chosen for its specific biological properties and relevance in tissue engineering and regenerative medicine approaches. Specifically, primary cells, including CMSCs and BM-MSCs, were chosen for their ability to closely mimic in vivo conditions, providing physiologically relevant insights. In contrast, cell lines were used for their reproducibility and ease of handling, making them suitable for initial evaluations and preliminary studies. Cell morphology, adhesion, viability, and proliferation on PETG were compared to those on conventional cell culture plastic.

#### 3.2.1. Morphological Analysis

**Figure 3:**
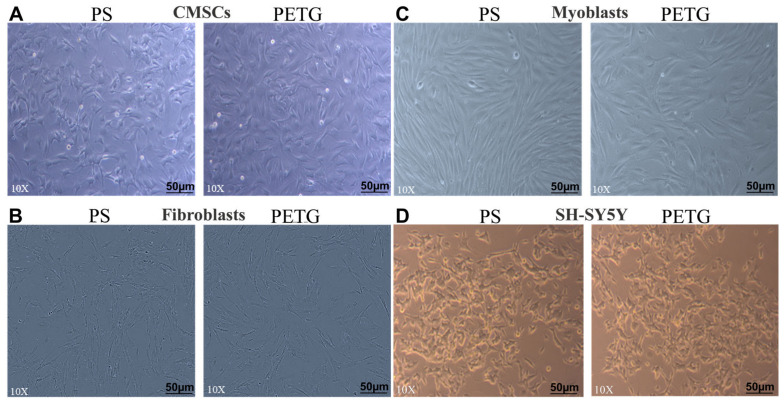
CMSCs, myoblasts, fibroblasts and SH-SY5Y culture on PS and PETG substrates. Representative images of cell morphology obtained using light microscopy at 10× magnification. (**A**) CMSCs, (**B**) fibroblasts, (**C**) myoblasts, and (**D**) SH-SY5Y cells were seeded on both PETG and PS substrates (serving as a standard for comparison) and cultured in proliferative medium for 72 h. Scale bars: 50 µm.

Morphological analysis using light microscopy provided qualitative insights into the effects of PETG culture substrates on cell morphology, with PS substrates serving as a reference for comparison. Our results showed that PETG did not significantly influence cell morphology regardless of the cell type (Figure 3). Among these, CMSCs grown on PETG for 72 h exhibited a typical spindle-shaped morphology with elongated, fibroblast-like structures and a uniform bipolar orientation, characteristic of undifferentiated MSCs, closely resembling that observed on PS (Figure 3A). Similarly, human dermal fibroblasts cultured on PETG maintained their characteristic elongated, spindle-like shape, with cells evenly distributed across the support surface and presenting a homogeneous phenotype after 72 h (Figure 3B). LHCN-M2 myoblasts cultured on both PETG and PS displayed an elongated, spindle-shaped morphology (Figure 3C), characteristic of undifferentiated myogenic precursors. The cells were aligned with minimal cytoplasmic extensions, consistent with the proliferative state of early myoblasts (Figure 3C). SH-SY5Y neuroblastoma cells cultured on PETG substrates presented a polygonal cell body with short, neurite-like processes, indicative of their undifferentiated and proliferative state, comparable to the morphology observed on PS (Figure 3D).

These findings highlighted that the morphological characteristics of the different cell types cultured on PETG substrates were fully overlapping with those grown on traditional PS substrates. These results supported PETG’s effectiveness as a biocompatible and reliable alternative to conventional laboratory plastics, making it a promising option for cell culture and other laboratory applications.

#### 3.2.2. Cell Adhesion Analysis

To quantitatively assess early CMSC adhesion on PETG substrates, a washing-based adhesion assay was performed on PS and gelatin-coated PETG substrates. This analysis was introduced to determine the percentage of viable adherent cells retained on each substrate after the removal of non-adherent or loosely attached cells. CMSCs were analyzed at early post-seeding time points, namely 6, 12, and 24 h.

**Figure 4:**
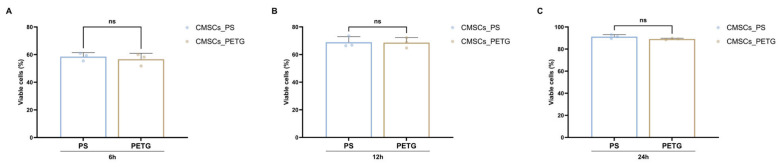
Quantitative assessment of CMSC adhesion after washing on PS and PETG substrates. CMSCs were seeded on PS and gelatin-coated PETG substrates and analyzed after 6 h (**A**), 12 h (**B**), and 24 h (**C**). At each time point, substrates were washed to remove non-adherent or loosely attached cells, and viable adherent cells recovered after washing were counted using AO/PI staining. Results are expressed as the percentage of viable adherent cells relative to the initial number of seeded cells. Data are presented as mean ± SD. Statistical significance between PS and PETG at each time point was assessed using an unpaired Student’s *t*-test; ns, not significant.

After washing, the percentage of viable adherent cells was comparable between PS and PETG at all analyzed time points. Specifically, no significant differences were observed between the two substrates at 6 h (Figure 4A), 12 h (Figure 4B), or 24 h (Figure 4C). These results indicate that gelatin-coated PETG supported early CMSC adhesion after washing similarly to conventional PS under the tested conditions.

**Figure 5:**
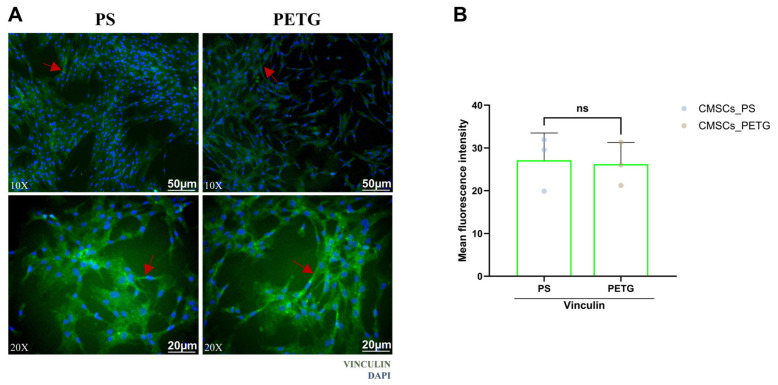
Immunofluorescence analysis of FAs in CMSCs cultured on PETG and PS substrates. (**A**) Representative fluorescence microscopy images of CMSCs seeded on PS and PETG and stained with anti-vinculin antibody (green) to visualize focal adhesions and DAPI (blue) to stain nuclei after 48 h of proliferation. Red arrows indicate the presence of FAs. Images were captured at 10× (top images) and 20× (bottom images) magnification; (**B**) Fluorescence intensity of vinculin staining was measured in CMSCs cultured on PETG and PS for 48 h in proliferation medium. Data are presented as mean (*n* = 3) ± SD, and statistical significance was determined using an unpaired Student’s *t*-test; ns: not significant. Scale bars: 50–20 µm.

**Figure 6:**
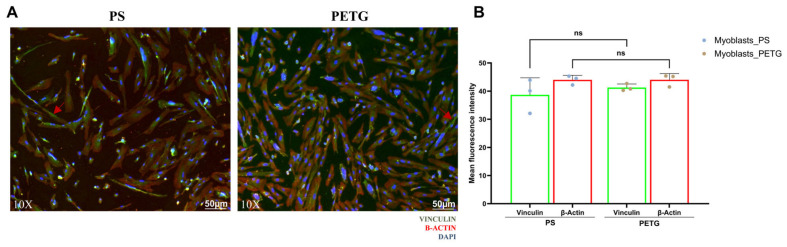
Immunofluorescence analysis of FAs in LHCN-M2 myoblasts. (**A**) Representative fluorescence microscopy images of myoblasts seeded on PS and PETG substrates, and stained with anti-vinculin antibody (green), anti-β-actin (red) and DAPI (blue) to detect FAs, the cytoskeleton, and nuclei, respectively. Images were captured at 10×. Red arrows indicate the presence of FAs; (**B**) Quantification of fluorescence intensity for vinculin and β-actin staining in LHCN-M2 cultured on PETG and PS for 48 h in proliferation medium. Data are presented as mean (*n* = 3) ± SD, and statistical significance was determined using one-way ANOVA; ns: not significant. Scale bars: 50 µm.

Focal adhesions (FAs) are crucial for mechanical stability and intercellular signaling, linking the extracellular matrix (ECM) to the intracellular cytoskeleton. Vinculin, a key FA protein, anchors the actin cytoskeleton to the cell membrane at adhesion sites, promoting cellular attachment and spreading [39,40]. To assess cellular interactions with PETG substrates and evaluate the establishment of cell cultures, vinculin distribution was analyzed in CMSCs (Figure 5) and LHCN-M2 myoblasts (Figure 6) cultured on PETG. Immunofluorescence analysis revealed that vinculin was prominently localized at focal adhesions, indicating stable cell attachment on both substrates (Figure 5A). The fluorescence microscopy images showed a comparable vinculin distribution between PETG and PS, suggesting that PETG supported cellular adhesion similarly to conventional culture plastics (Figure 5A). Fluorescence intensity analysis confirmed that CMSCs cultured on PETG exhibited vinculin levels comparable to those observed in cells seeded on PS (Figure 5B).

This analysis provided insights into how PETG supported focal adhesion plaque formation and influenced cell attachment dynamics.

To further investigate FA dynamics, the distribution of vinculin and β-actin organization in myoblasts cultured on PS and PETG substrates was analyzed. β-actin was included owing to its crucial role in cytoskeletal organization and mechanotransduction, as reported in the literature [41]. Immunofluorescence analysis revealed a well-organized vinculin distribution at FAs, co-localizing with β-actin filaments. This suggested that PETG substrates supported cytoskeletal organization and cell adhesion similarly to standard PS substrates (Figure 6A). Furthermore, cell spreading profiles were consistent between PETG and PS (Figure 6A), reinforcing the idea that PETG effectively supported cell attachment and cytoskeletal stability, comparable to standard plastic. Fluorescence intensity quantification indeed revealed no significant differences in vinculin levels between myoblasts cultured on PETG and those on PS. Likewise, β-actin intensity was comparable across both substrates, suggesting that the material composition did not significantly affect the overall expression of these proteins.

This result underscored PETG’s ability to provide an environment conducive to proper cellular function through efficient cell–substrate interactions.

#### 3.2.3. Cell Viability Assay

**Figure 7:**
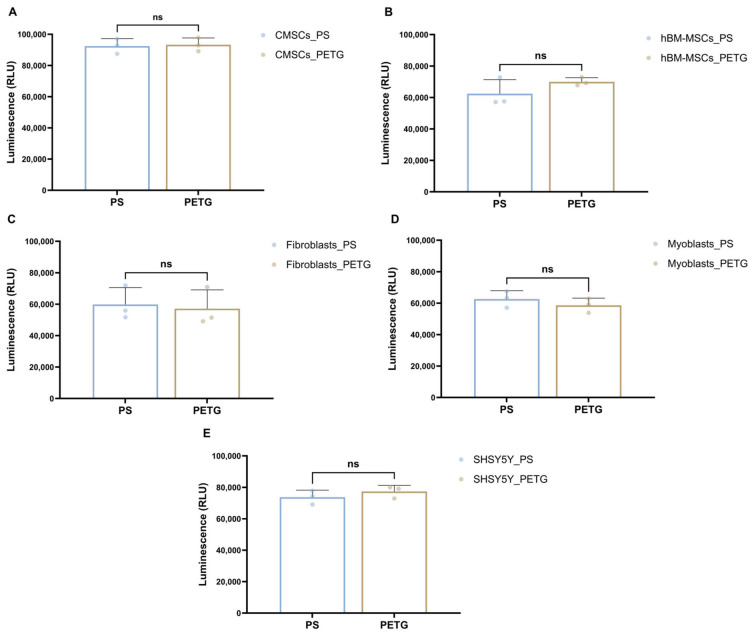
Cell viability assay on PETG and PS substrates. Bar graphs depict the viability of (**A**) CMSCs, (**B**) hBM-MSCs, (**C**) fibroblasts, (**D**) myoblasts, and (**E**) SH-SY5Y after 48 h of culture on standard PS (blue bar) and PETG (yellow bar) substrates with proliferative medium. Cell viability was assessed using the CellTiter-Glo^®^ assay, which measures luminescence (RLU) as an indicator of metabolically active cells. Data are presented as mean (*n* = 3) ± SD, and statistical significance was determined using an unpaired Student’s *t*-test; ns: not significant.

Cell viability on PETG was evaluated using the CellTiter-Glo^®^ assay, which quantifies adenosine triphosphate (ATP) levels as an indicator of metabolically active cells. CMSCs, hBM-MSCs, fibroblasts, myoblasts, and SH-SY5Y cultured on PETG for 48 h exhibited luminescent signal levels similar to those observed on standard laboratory plastic, with no statistically significant differences detected (Figure 7A–E). In each condition analyzed, luminescence values consistently ranged between 60,000 and 100,000 RLU (Relative Luminescence Units), indicating similar metabolic activity and, consequently, comparable cell viability between PETG and conventional PS.

The cell viability results confirmed that PETG met the critical requirement of biocompatibility. As a bioinert material, it did not induce evident cytotoxic effects under the tested conditions, thus providing a stable and supportive environment essential for sustaining metabolic activity and avoiding cytotoxic effects, which are key conditions for promoting cell adhesion, proliferation, and differentiation in experimental biology studies [41].

#### 3.2.4. Cell Proliferation on PETG

##### Live-Cell Analysis

**Figure 8:**
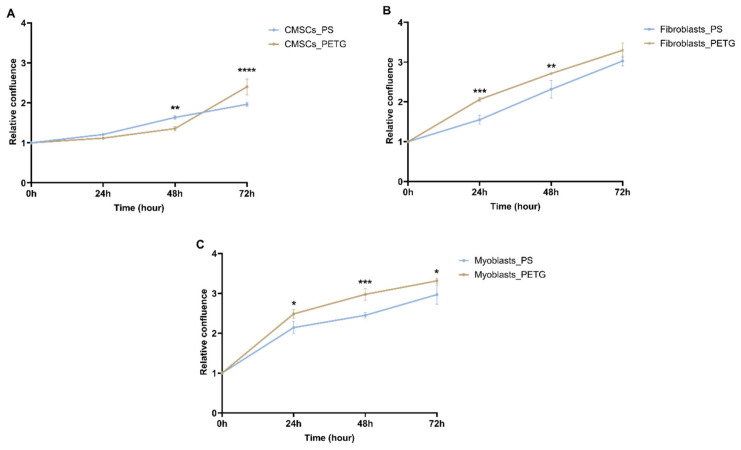
Cell proliferation on PETG and PS substrates. Proliferation rate of (**A**) CMSCs, (**B**) fibroblasts, and (**C**) myoblasts grown on PETG (yellow curve) and standard PS (blue curve) control supports over a 72h period using Live-Cell Analysis. Proliferation rate on each surface was determined by calculating the confluence ratio, comparing the cell-covered area at the initial time point (hour 0) of cell seeding on substrates with the area covered by cells at different post seeding time points (24, 48 and 72 h). Data are presented as mean (*n* = 3) ± SD, and statistical significance was determined using two-way ANOVA comparing PETG and PS at each time point (24, 48, 72 h). Significance levels are indicated as *p* ≤ 0.05 (*); *p* ≤ 0.01 (**); *p* ≤ 0.001 (***); *p* ≤ 0.0001 (****).

Proliferation analysis was conducted on CMSCs, fibroblasts, and myoblasts seeded on PETG and compared to control plastic substrates. Live imaging analysis revealed that CMSCs grown on both PETG and PS substrates showed an increasing proliferation trend over 72 h (Figure 8A). Of note, CMSCs on PS exhibited a significantly higher proliferative potential compared to those on PETG after 48 h of incubation (Figure 8A). This shift in proliferation dynamics during the later phase of culture (48 to 72 h) (Figure 8A), may indicate distinct temporal responses of CMSC growth, depending on the culture substrate.

Fibroblasts cultured on PETG showed a significantly higher proliferation rate at 24 and 48 h of incubation compared to cells grown on PS, with this trend persisting, although less pronounced, at 72 h (Figure 8B). Similarly, myoblasts cultured on PETG exhibited significantly increased cell proliferation compared to those cultured on PS at all time points analyzed (Figure 8C).

These analyses demonstrated PETG’s capacity to support consistent growth of different cell types, which was even more pronounced compared to PS, suggesting its ability to provide a stable and conducive microenvironment for cellular activities, such as nutrient absorption, matrix remodeling, and cell–cell interactions.

##### Gene Expression Profile of CMSCs on PETG

**Figure 9:**
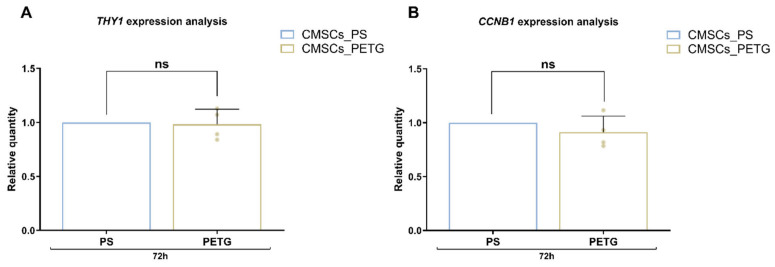
Gene expression analysis of stemness and proliferation markers in CMSCs on PETG and PS. (**A**,**B**) Gene expression analysis of *THY1* and *CCNB1* genes in CMSCs cultured on PETG and PS for 72 h in proliferative medium. Data are presented as mean (*n* = 4) ± SD, and statistical significance was assessed using an unpaired Student’s *t*-test; ns: not significant.

To characterize the expression profiles of stemness and proliferation markers of CMSCs cultured on PETG, the expression levels of *THY1* (*THY-1-1 cell surface antigen*) and *CCNB1* (*Cyclin B1*) genes were analyzed by qPCR. *THY1*, a recognized marker of undifferentiated CMSCs [42], showed comparable expression levels in cells cultured on both PETG and PS supports for 72 h under proliferative conditions (Figure 9A). Similarly, expression analysis of *CCNB1*, a critical regulator of cell cycle progression and proliferation [43,44,45,46], indicated no significant differences between CMSCs grown on PETG and PS (Figure 9B).

Overall biocompatibility analysis demonstrated that PETG supported the proliferation of CMSCs while maintaining transcriptomic profiles comparable to those observed with conventional substrates (Figure 9), including the stable expression of key markers such as *THY1*, crucial for maintaining stemness. This aligns with our previous research [33], which emphasized the significance of maintaining the stable expression of stemness markers such as *THY1* and proliferation-associated genes to preserve the functional properties of CMSCs during in vitro culture.

### 3.3. Evaluation of Cell Differentiation on PETG

The evaluation of PETG’s capacity to sustain various cell differentiation pathways, including osteogenic, adipogenic, neuronal, and myogenic differentiation, was pivotal for understanding its potential as a cell culture substrate in laboratory practices.

#### 3.3.1. Osteogenic Differentiation of CMSCs on PETG

**Figure 10:**
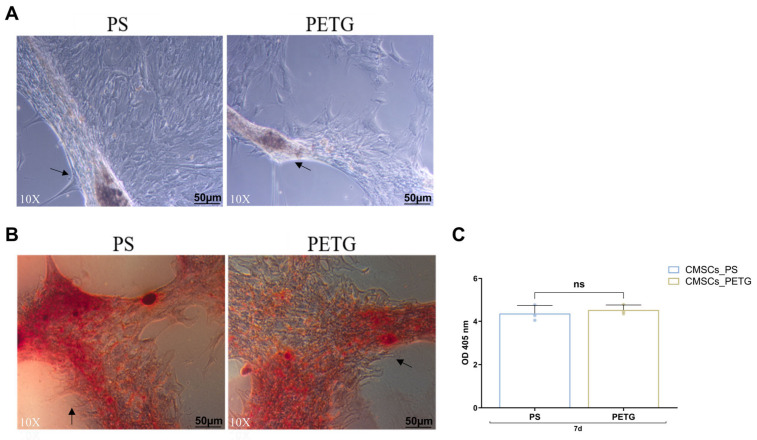
Osteogenic differentiation of CMSCs on PETG and PS. (**A**) Representative images of differentiated CMSCs obtained using light microscopy (10×). CMSCs were seeded on both PETG and PS substrates (control) and cultured in osteogenic medium for 7 days. Black arrows indicate the presence of mineralized matrix; (**B**) Representative light microscopy images (10× magnification) of CMSCs stained with Alizarin Red S (ARS) after 7 days of osteogenic induction. Black arrows indicate the presence of mineralized matrix, which stained red due to the presence of calcium ions; (**C**) The graph displays the quantification of ARS-stained bone mineralized matrix deposited by cells cultured on PETG and PS under osteogenic conditions for 7 days. Graph bars represent the optical density (OD) mean values (*n* = 3) ± SD. Results were analyzed by unpaired Student’s *t*-test; ns: not significant. Scale bars: 50 µm.

To ascertain whether PETG offers a comparable microenvironment to PS in promoting osteogenesis, CMSCs were induced to differentiate into pre-osteoblasts/osteoblasts. Morphological analysis revealed that cells cultured on PETG exhibited a pre-osteoblastic phenotype and deposited a mineralized bone matrix like those observed in cells cultured on control PS after 7 days of osteogenic induction (Figure 10A). These findings were corroborated by Alizarin Red S staining (Figure 10B) and its quantification (Figure 10C), which demonstrated comparable levels of stained bone mineralized matrix deposited by cells on both substrates (Figure 10B,C). These results confirmed that CMSCs on PETG and PS supported matrix deposition to an equivalent extent, underscoring PETG’s suitability for supporting the osteogenic differentiation of CMSCs (Figure 10).

**Figure 11:**
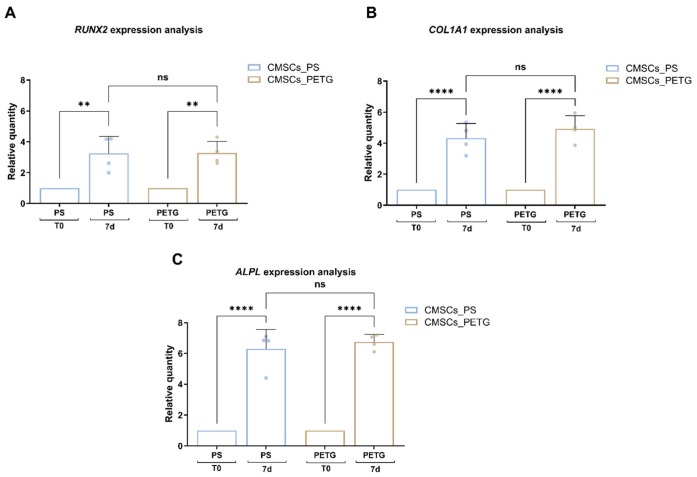
Expression analyses of osteogenic markers during osteogenic differentiation of CMSCs. Relative transcript levels of key osteogenic markers such as *RUNX2* (**A**), *COL1A1* (**B**) and *ALPL* (**C**) in CMSCs cultured on PETG and PS with osteogenic medium for 7 days (7 d). Cells grown on both substrates under proliferative conditions were used as controls (T0). Data are presented as mean (*n* = 4) ± SD, and statistical significance was determined using one-way ANOVA; *p* ≤ 0.01 (**); *p* ≤ 0.0001 (****); ns: not significant.

Levels of key osteogenic markers such as *RUNX2* (*runt-related transcription factor 2*), *COL1A1* (*collagen type I alpha 1*), and *ALPL* (*alkaline phosphatase*, *biomineralization associated*), were analyzed to further assess the osteogenesis of CMSCs on PETG compared to PS. A significant upregulation in the expression of the aforementioned markers was observed in cells cultured on each substrate for 7 days in the osteogenic medium compared to undifferentiated cells (T0), confirming the successful osteogenic commitment of CMSCs on both supports (Figure 11A–C). Notably, *RUNX2* (Figure 11A), *COL1A1* (Figure 11B), and *ALPL* (Figure 11C) levels were comparable between cells grown on PETG and PS substrates in control cells (T0) and after 7 days of osteogenic induction (Figure 11A–C; Supplementary Figure S3).

Our results confirmed PETG’s relevance in bone tissue engineering applications, consistent with the existing literature. In this context, Hassan and collaborators demonstrated that PETG scaffolds provided a biomechanical environment conducive to enhanced cell attachment and proliferation [39]. In their study, PETG scaffolds with a 0/90 lay-down pattern and varying pore sizes (300, 350, and 450 µm) were fabricated using a filament-based extrusion additive manufacturing system and subsequently subjected to mechanical and biological characterization. The results showed that PETG scaffolds exhibited significantly higher mechanical properties compared to polycaprolactone (PCL) scaffolds. Furthermore, cytotoxicity assessments using human adipose-derived stem cells (hADSCs) confirmed the biological compatibility of PETG scaffolds, highlighting their potential as a reliable material for bone scaffold fabrication [39].

#### 3.3.2. Adipogenic Differentiation of hBM-MSCs on PETG

**Figure 12:**
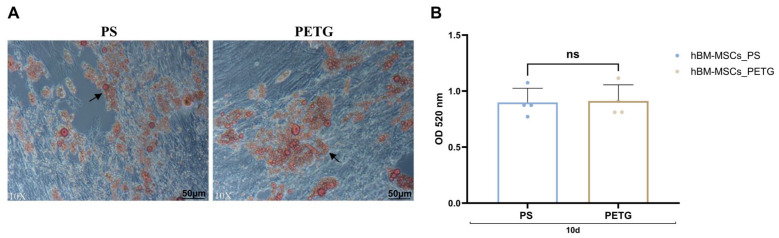
Adipogenic differentiation of hBM-MSCs on PETG and PS. (**A**) Representative light microscopy images (10×) of Oil Red O (ORO) staining of lipid droplets (in red; black arrows) in hBM-MSCs induced to adipogenic differentiation on PS and PETG substrates for 10 days; (**B**) Quantitative analysis of ORO-stained lipid droplets in hBM-MSCs cultured on PETG and PS under adipogenic conditions for 10 days. Optical density (OD) measurements of ORO were used to assess and compare lipid accumulation in hBM-MSCs cultured on both supports. Data are presented as mean (*n* = 4) ± SD, and statistical significance was determined using an unpaired Student’s *t*-test; ns: not significant. Scale bars: 50 µm.

To evaluate the ability of PETG to sustain a broad range of cell differentiation pathways, adipogenic differentiation on PETG substrates was also analyzed and compared with conventional PS culture substrates. To this aim, hBM-MSCs were induced toward adipogenic differentiation for 10 days. To evaluate the differentiation of hBM-MSCs into maturing pre-adipocytes/mature adipocyte, ORO staining—a lipophilic agent—was used to stain lipid droplets within cells, which is a hallmark of adipogenic differentiation (Figure 12A). Stained lipid droplets were then quantified to assess and compare their levels in cells cultured on PETG and PS (Figure 12B). Our data revealed comparable levels of adipocyte formation between cells grown on PETG and PS (Figure 12A,B), with OD values ranging between 0.5 and 1.5 on both substrates (Figure 12B). The quantification of stained lipid droplets in hBM-MSCs confirmed PETG’s capacity to support adipogenic differentiation, comparable to conventional plastic. Additionally, it demonstrated PETG’s compatibility with standard laboratory assays, including ORO staining and quantification tests.

#### 3.3.3. Myogenic Differentiation of LHCN-M2 Human Myoblasts on PETG

**Figure 13:**
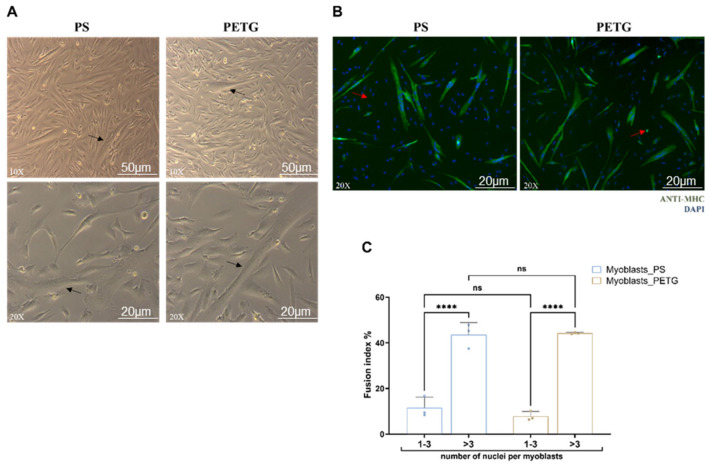
Assessment of myogenic differentiation by immunofluorescence. (**A**) Representative images (10× and 20×) of myoblasts seeded on PETG and PS, showing early myogenic differentiation after 72 h of induction. Black arrows indicate the formation of myotubes in cell culture; (**B**) Representative images of myoblasts cultured on PETG and PS after 7 days of myogenic differentiation and stained with anti-MHC antibody (green) to label muscle fibers and DAPI (blue) for nuclei. Red arrows indicate areas of myotube formation. Magnification: 20×; (**C**) Fusion index was calculated at the end of 7 days of myogenic differentiation as the ratio of the number of nuclei in the myotubes to the total number of nuclei in 12 randomly selected fields per condition from three independent experiments. Data are presented as mean (*n* = 3) ± SD, and statistical significance was determined using one-way ANOVA. Significance levels are indicated as *p* ≤ 0.0001 (****); ns: not significant. Scale bars: 50–20 µm.

The potential of PETG to sustain the myogenic differentiation of LHCN-M2 human myoblasts was also evaluated. During the 7-day induction period with the myogenic differentiation medium, myoblasts on PETG were monitored to identify key morphological changes associated with early and advanced myogenic differentiation. The early stages of differentiation, as shown in Figure 13A, were marked by the formation of myotubes, multinucleated syncytia, and fusing myoblasts on both conventional plastic substrates and PETG.

To further validate myogenic differentiation and investigate the effect of the PETG substrate on cell commitment, immunofluorescence staining and gene expression analysis were performed. Specifically, immunofluorescence staining for myosin heavy chain (MHC) allowed calculation of the fusion index, defined as the ratio of MHC-positive nuclei within myotubes to the total number of nuclei per field (with myotubes defined as containing three or more nuclei) (Figure 13B,C). The results showed no statistically significant differences in fusion indices between myoblasts cultured on PETG and those on PS. Notably, both groups exhibited a significant increase in myotubes containing more than 4 nuclei compared to unfused myoblasts, confirming effective myogenic differentiation on both PETG and PS substrates (Figure 13C).

**Figure 14:**
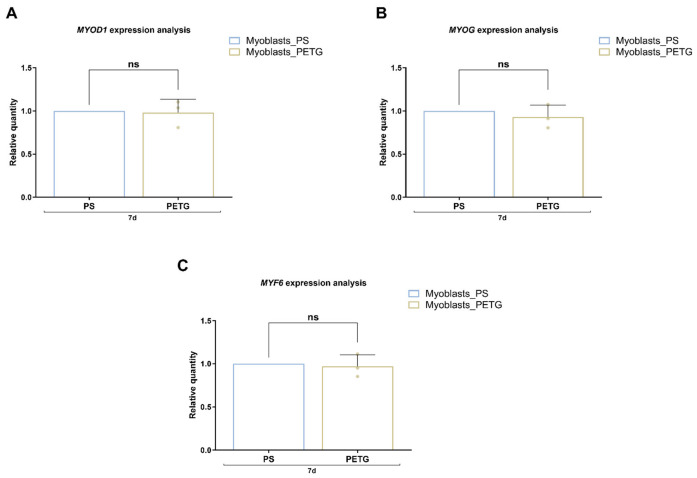
Gene expression analysis of myogenic markers in differentiated LHCN-M2 on PETG and PS. Expression levels of key myogenic markers, (**A**) *MYOD1*, (**B**) *MYOG*, and (**C**) *MYF6*, were evaluated in myoblasts cultured on PETG and PS following myogenic differentiation for 7 days. Data are presented as mean (*n* = 3) ± SD, and statistical significance was assessed using an unpaired Student’s *t*-test; ns: not significant.

Moreover, gene expression analysis of myogenic markers, including *MYOD1* (*myoblast determination protein 1*), *MYOG* (*myogenic*), and *MYF6* (*myogenic factor 6*), showed comparable levels between myoblasts cultured on PETG and PS for 7 days under myogenic conditions (Figure 14A–C). These data confirmed PETG’s ability to support myotube formation, making it a viable material for in vitro myogenic applications.

#### 3.3.4. Neuronal Differentiation of SH-SY5Y Neuroblastoma Cells on PETG

**Figure 15:**
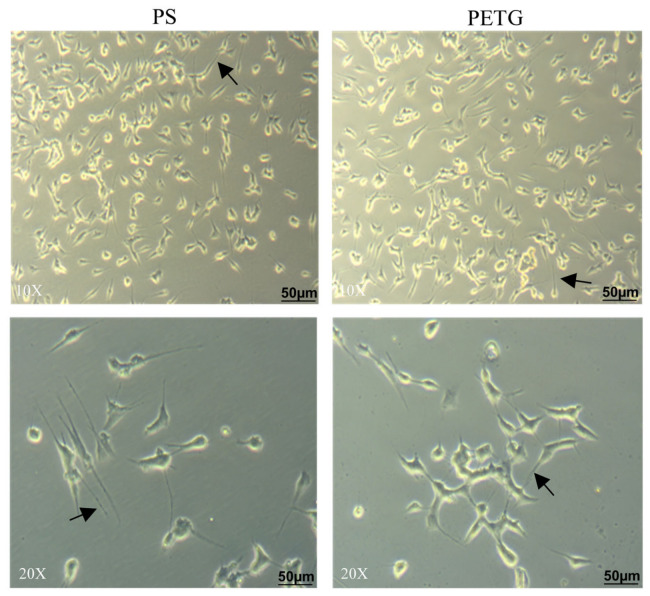
**Neuronal differentiation of SH-SY5Y cells on PS and PETG.** Representative images of differentiated SH-SY5Y were obtained using light microscopy at 10× (top images) and 20× (bottom images) magnification. SH-SY5Y were seeded on PETG and PS (control) and cultured in differentiation medium containing retinoic acid and B27 supplements for 10 days. Black arrows indicate the presence of elongated neuritic projections of differentiated cells.

Neuronal differentiation was evaluated to determine whether PETG could support the commitment of neuroblastoma cells into a neuronal lineage. After 10 days of differentiation with RA and B27 supplements, SH-SY5Y neuroblastoma cells displayed a neuronal morphology, characterized by the development of elongated neuritic projections, which is a key indicator of neuronal differentiation (Figure 15). The cell bodies of SH-SY5Y cells, along with the interconnected network of neurites, were clearly visible in phase-contrast images captured at 10× and 20× magnification (Figure 15). Comparative analysis of SH-SY5Y cells cultured on PETG versus conventional PS showed no significant differences in morphology or neurite outgrowth.

**Figure 16:**
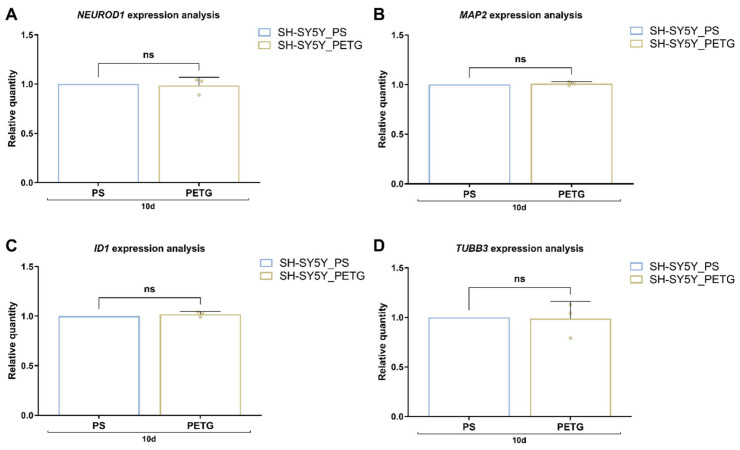
Expression analyses of neuronal markers during neuronal differentiation of SH-SY5Y cells. Relative transcript levels of key neuronal markers such as *NEUROD1* (**A**), *MAP2* (**B**), *ID1* (**C**) and *TUBB3* (**D**) in SH-SY5Y cells cultured on PETG compared to those cultured on PS with neuronal differentiation factors (RA and B27) for 10 days (10 d). Data are presented as mean (*n* = 3) ± SD, and statistical significance was assessed using an unpaired Student’s *t*-test; ns: not significant.

To further validate the occurrence of neuronal differentiation on both substrates, gene expression analysis was performed for key neuronal markers, including *NEUROD1* (*neurogenic differentiation 1*), *MAP2* (*microtubule-associated protein 2*), *ID1* (*inhibitor of DNA binding 1*), and *TUBB3* (*tubulin beta 3 class III*). Comparable expression levels were observed in SH-SY5Y cells cultured on PETG and PS for 10 days under neurogenic conditions (Figure 16A–D), confirming that PETG is equally effective as PS in supporting neuronal commitment at the molecular level.

These results suggested that PETG effectively supported neuronal differentiation, demonstrating its potential to facilitate this process at a level comparable to conventional PS. Considering that neuronal differentiation is generally more complex and challenging than other cell commitment processes, PETG’s ability to sustain this intricate process further highlighted its suitability for applications in neural tissue engineering and other neuronal studies.

### 3.4. Reuse of PETG Substrates

The development and implementation of reusable materials in biomedical applications align with the principles of the circular economy, addressing pressing environmental and economic challenges. Among polymers widely employed in cell culture, PETG offers a distinct advantage over PS due to its 100% recyclability. Unlike PS, which is difficult to recycle effectively and often destined for landfills, PETG can be reprocessed into new materials with minimal loss of mechanical properties. These characteristics make PETG an ideal candidate for sustainable practices in medical and research settings, where the reliance on single-use plastics remains significant.

PETG substrates, produced as previously described, were also tested to evaluate their potential for repeated use in cell culture systems. In our analysis, PETG-based substrates were sterilized and reused directly without undergoing re-extrusion or additional material processing to assess whether the supports, after decontamination, could maintain the necessary structural and functional properties required for effective cell culture. Thus, PETG substrates were recovered after the cell culture experiments described above. The sterilization of PETG followed a multi-step procedure designed to ensure no visible residual cellular material was detected by light microscopy. A final microscopic inspection verified the absence of visible residues, confirming the supports’ readiness for reuse in cell culture studies.

Current research has focused on biocompatible and biodegradable materials, such as polycaprolactone (PCL), polylactic-co-glycolic acid (PLGA), starch-based polymers, and polybutylene succinate (PBS), due to their sustainability benefits across various applications [47]. However, despite their advantages, challenges related to biodegradability and sterilization, particularly with PCL and PLGA, have hindered their use in certain laboratory settings. Indeed, the degradation of these materials over time can complicate long-term storage and handling, particularly when subjected to repeated sterilization cycles (e.g., exposure to heat, radiation, or chemical agents) [48,49]. This degradation can interfere with the stability and consistency required for standardized laboratory protocols, especially in critical applications such as culture supports, where maintaining material integrity is essential. In contrast, while PETG is not biodegradable, its durability, recyclability, and compatibility with low-temperature recovery/sterilization procedures make it a promising candidate for repeated-use laboratory applications. In the present study, this potential was supported by short-term reuse experiments showing preserved CMSC morphology and metabolic activity after repeated reuse under the tested conditions.

#### 3.4.1. Morphological Comparison of CMSCs on Reused PETG vs. Single-Use PETG

**Figure 17:**
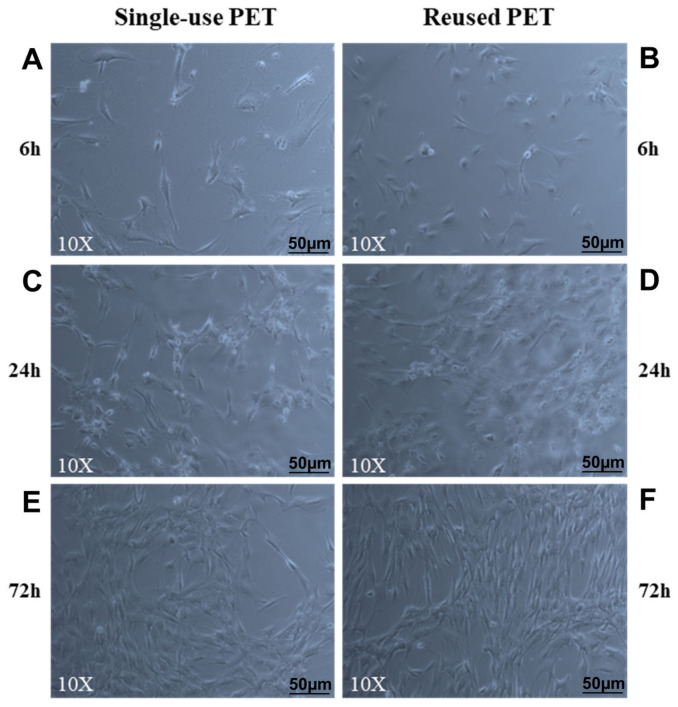
Morphology of CMSCs cultured on single-use and reused PETG substrates. Representative light microscopy images of CMSCs cultured under proliferative conditions on single-use PETG and reused PETG for 6 h (**A**,**B**), 24 h (**C**,**D**), and 72 h (**E**,**F**). Scale bars: 50 µm.

Following sterilization, reused PETG substrates were tested for their ability to support the growth of CMSCs. Specifically, comparative analyses were performed to evaluate the differences between CMSCs cultured on reused PETG and those cultured on single-use PETG. Morphological analysis by light microscopy revealed no evident morphological differences in the CMSC phenotype between cells cultured on reused PETG and those on single-use PETG. Specifically, the assessment of cell adhesion and morphology was conducted at 6, 24- and 72 h post-seeding of CMSCs on both single-use PETG and reused PETG (Figure 17A–F). After 6 h, the newly adhered cells began to adhere similarly to both substrates. (Figure 17A,B). By 24 h, the cells exhibited the characteristic fibroblast-like morphology, indicating that adhesion and spreading had occurred correctly on single-use and reused PETG, according to literature data (Figure 17C,D). Furthermore, after 72 h of cell incubation, the morphological analysis indicated an increase in cell numbers, suggesting active proliferation on both supports (Figure 17E,F). These results confirmed that reused PETG was as effective as single-use PETG in facilitating cell adhesion, spreading, and growth, without apparently compromising cell viability or morphology.

#### 3.4.2. Cell Viability After Repeated PETG Reuse Cycles

To further investigate the effect of repeated reuse on PETG performance, CMSCs were seeded on PETG substrates with different reuse histories and analyzed after 48 h of culture. Non-reused PETG substrates were used as controls and compared with PETG substrates reused once, twice, or three times, defined as PETG-new, PETG-R1, PETG-R2, and PETG-R3, respectively.

**Figure 18:**
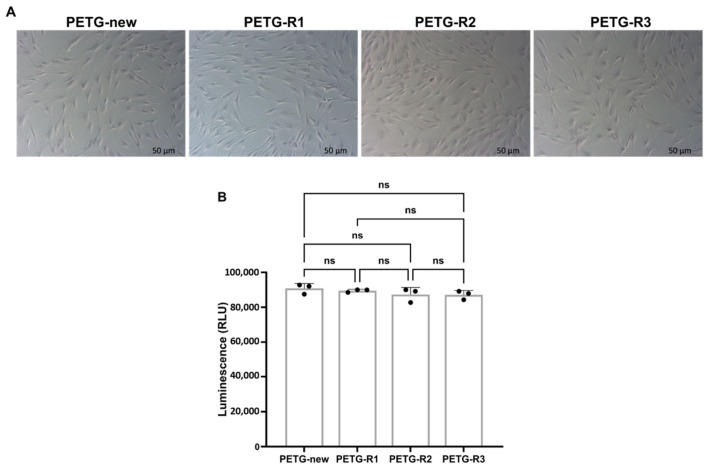
CMSCs morphology and metabolic activity on PETG substrates after repeated reuse cycles. Representative light microscopy images (10×) showing CMSCs morphology after 48 h of culture on PETG substrates subjected to 0, 1, 2, or 3 reuse cycles, corresponding to PETG-new, PETG-R1, PETG-R2, and PETG-R3, respectively (**A**). Cell viability/metabolic activity was assessed at the same time point by CellTiter-Glo^®^ assay and reported as absolute RLU (**B**). Data are presented as mean ± SD (*n* = 3); statistical analysis was performed using one-way ANOVA followed by Tukey’s multiple-comparison test; ns, not significant. Scale bars: 50 µm.

At the end of each 48 h culture period, representative light microscopy images were acquired before CellTiter-Glo^®^ analysis to document CMSC adhesion and morphology under each condition. CMSCs maintained an adherent, elongated morphology on all tested substrates, including PETG-R3 (Figure 18A). CellTiter-Glo^®^ analysis showed no significant differences in absolute luminescence values among PETG-new, PETG-R1, PETG-R2, and PETG-R3 (Figure 18B). These results indicate that PETG reuse up to the third cycle did not significantly impair CMSC viability/metabolic activity after 48 h of culture under the tested conditions.

Overall, these data extend the preliminary single-reuse observations and support the short-term, repeated reuse feasibility of PETG substrates. However, further studies will be required to evaluate a higher number of reuse cycles, longer culture durations, additional cell types, sterility, endotoxin levels, leachable release, and detailed surface, optical, and mechanical properties after repeated reuse.

Our preliminary data support the reuse potential of PETG substrates, suggesting a possible alignment with circular economy principles. The ability to recover, sterilize, re-coat, and reuse PETG substrates while maintaining cell adhesion, morphology, and viability/metabolic activity under the tested short-term conditions represents an encouraging step toward more sustainable laboratory practices. The comparable performance observed between non-reused PETG and PETG reused up to three times further supports its potential as a functional substrate for cell culture applications.

## 4. Conclusions

This study highlights the potential of PETG as a biocompatible and potentially reusable substrate for routine two-dimensional cell culture applications. Although PETG has been previously explored in biomedical and 3D-printing-related applications, its use as a laser-cut, optically transparent, flat sheet substrate for standard cell culture workflows has been less investigated. In this context, the present study focuses on PETG not as a generic biomaterial, but as a practical culture support combining a simple laser-cut substrate format, multi-cell-type biological evaluation, and a preliminary short-term repeated-reuse assessment.

Recent advancements in sustainable laboratory consumables have introduced biodegradable and bio-based plastics for pipette tips, tubes, and culture plates. Companies such as Corning^®^, Greiner Bio-One^®^, and Eppendorf^®^ have developed labware using recycled polypropylene (rPP), PLA-based bioplastics, and refillable systems to reduce single-use plastic waste. For example, Eppendorf has launched the Eppendorf Consumables BioBased line, which includes epT.I.P.S.^®^ BioBased pipette tips and twin.tec^®^ PCR Plates BioBased, made from second-generation renewable raw materials such as waste oils [49]. Similarly, Greiner Bio-One offers 96-well polypropylene microplates, promoting the use of more sustainable plastics in laboratory applications [50]. Meanwhile, Corning Life Sciences is pioneering advanced chemical recycling technologies to convert used laboratory plastics into high-purity raw materials suitable for new labware production [51]. While these innovations represent a positive step toward greener laboratory practices, several challenges still limit their widespread implementation. Studies have highlighted key issues such as limited sterilization stability, mechanical fragility, and high production costs. Biodegradable plastics, such as PLA, are highly sensitive to standard laboratory sterilization processes, which can cause thermal and hydrolytic degradation, leading to structural damage, chemical alterations, and potential toxic by-product release, ultimately limiting their usability in biomedical applications [52]. Moreover, many bioplastics exhibit low mechanical strength and thermal resistance, limiting their feasibility for routine laboratory use [53]. Given these drawbacks, there is a growing need for durable and stable alternatives that can be compatible with routine cell culture workflows and potentially reused in laboratory environments.

Recyclable thermoplastics such as PETG may provide a promising material platform for cell culture applications, potentially supporting both experimental reproducibility and more sustainable laboratory practices. PETG has a well-established mechanical recycling infrastructure, with studies suggesting that recycled PETG can retain its biocompatibility and structural integrity after multiple reuse cycles [54,55,56]. Compared with conventional single-use PS culture plastics, PETG combines optical transparency, chemical resistance, durability, and recycling/reprocessing potential, making it a promising candidate for the development of reusable culture substrates and circular economy-oriented laboratory workflows [54,55,56,57].

Compared with reusable or semi-reusable culture systems such as glass coverslips, glass cultureware, glass-bottom devices, or silicone/PDMS-based platforms, laser-cut PETG may offer practical advantages related to lightweight handling, optical transparency, low-cost fabrication, and customization into different cell culture formats and geometries. Nevertheless, direct benchmarking against commercially available reusable systems was not performed in the present study and should be considered in future comparative analyses.

In this study, PETG-based cell culture supports effectively sustained cell adhesion, proliferation, and differentiation across different cell types, including mesenchymal stromal cells (CMSCs and hBM-MSCs), fibroblasts, myoblasts, and neuroblastoma cells. Under the tested conditions, gelatin-coated PETG showed biological performance comparable to PS, supporting its suitability for in vitro applications requiring the preservation of cellular viability, morphology, and functionality. PETG also demonstrated compatibility with essential molecular and cell biology techniques, including viability assays, live-cell proliferation analysis, RNA extraction, qPCR, morphological assessment by optical and fluorescence microscopy, general staining procedures, and immunofluorescence analysis. Interestingly, the proliferation response to PETG differed among the analyzed cell types, with fibroblasts and myoblasts showing higher proliferation on gelatin-coated PETG compared with PS, whereas CMSCs displayed a different trend. This suggests a cell-type-specific response to the PETG–gelatin interface, possibly influenced by substrate-related cues such as surface morphology, wettability, protein adsorption, and material stiffness, which can affect adhesion, spreading, cytoskeletal organization, and mechanotransduction. PETG and PS may also differ in the adsorption, conformation, or organization of proteins at the culture interface, potentially affecting integrin engagement and focal adhesion formation. Although protein adsorption and mechanotransduction-related parameters were not directly assessed in this study, gelatin-coated PETG supported adhesion, proliferation, and lineage-specific differentiation comparably to PS under the tested conditions, suggesting that any potential differences did not evidently impair the main biological outcomes evaluated here.

Moreover, PETG’s short-term repeated reuse feasibility supports its potential to contribute to the reduction in single-use plastic waste in biomedical research. However, its overall sustainability profile compared with conventional culture plastics remains to be quantitatively assessed through dedicated life-cycle, cost, waste-reduction, energy-consumption, and end-of-life analyses. Therefore, PETG should be considered as a promising material for the development of more sustainable cell culture workflows, rather than as a quantitatively demonstrated sustainable replacement for conventional culture plastics.

Beyond direct reuse, future studies could investigate whether PETG substrates, after fulfilling their primary function in cell culture, could undergo mechanical re-extrusion and remanufacturing into new culture supports, extending their life cycle and further minimizing laboratory plastic waste. The feasibility of re-extruding PETG while preserving its biocompatibility and structural properties should therefore be further evaluated as a possible step toward sustainable laboratory practices. Currently, the majority of laboratory plastic waste is either incinerated or sent to landfills due to biosafety constraints, limiting the impact of traditional recycling efforts. Integrating PETG into circular economy strategies through enhanced recycling efficiency and closed-loop production cycles could offer a practical route to help reduce plastic waste in biomedical research.

Looking ahead, the present findings support the short-term feasibility of laser-cut PETG substrates for cell culture applications and identify key aspects that could be further investigated to support broader implementation. Since the main biological experiments were performed on gelatin-coated PETG substrates, future work may compare uncoated and differently coated PETG surfaces to better define the relative contribution of the material and of the adsorbed coating layer to cell behavior. In addition, further physicochemical analyses, including water contact angle, quantitative roughness, FTIR/XPS surface chemistry, surface charge, protein adsorption, and stiffness measurements, would help clarify the relationship between PETG surface properties and cellular responses. Longer-term studies involving higher numbers of reuse cycles will also be useful to assess mechanical integrity, optical transparency, surface stability, potential microstructural changes, leachable release, sterility, endotoxin levels, and the effects of repeated ethanol/UV treatment under different cell culture workflows. These future investigations will help define the long-term robustness, safety, sustainability, and practical applicability of PETG-based reusable culture substrates.

## Figures and Tables

**Table 1:** Manufacturer-reported composition and technical properties of Mayku Clear Sheet 0.5 mm PETG substrates.

**Raw Material Composition–PETG**				
**Material name**	**Specification (mm)**	**Contains chemical substance**	**CAS Number**	**Percentage**
PETG	0.2–1.5	Ethylene Glycol (EG)	107-21-1	18%
PETG	0.2–1.5	Terephthalic Acid (TPA)	100-21-0	77%
PETG	0.2–1.5	1,4-Cyclohexanedimethanol (CHDM)	105-08-8	5%
**PETG Sheet Ingredients (Mixed)**				
**Product**	**Material**	**Composition**		
Transparent PETG sheet	PETG	Original rubber particles, slip agent		
Blue PETG sheet	PETG	Original rubber particles, slip agent, blue dye		
White PETG sheet	PETG	Original rubber particles, slip agent, white pigment		
**Physical and Mechanical Performance**				
**Item**	**Standard**	**Unit**	**Value**	
Density	ASTM D792	g/cm^3^	1.27	
Impact strength	ASTM D256	J/m	90 (9.2)	
Heat resistant temperature	ASTM D648	°C	Humidity 35%, up to 60°C	
Deformation temperature	ASTM D648	°C	70	
Tensile strength	ASTM D638	MPa	26 (266)	
Tensile elongation at break	ASTM D638	%	140	
Haze	ASTM D1003	%	<10	
Transmittance	ASTM D1003	%	89	
**PETG Sheet Properties and Uses**				
**Item**	**Value**			
Density	1.27–1.30 g/cm^3^			
Melting point	220–260°C			
Glass transition temperature	78°C			
Colour	Colourless transparent; blue transparent; white			
Thickness	0.2–1.5 mm			
Width	460–760 mm			
Exterior	Flat, no air bubbles, no creases, no foreign matter			
Lightfastness	Yellowing after prolonged UV exposure			
Use	Used for blistering, folding boxes, printing, packaging, etc.			
Storage/use condition	Dry, dust-free, cool, protected from light and UV			

**Table 2:** Forward (F) and reverse (R) primer sequences used for real-time PCR (qPCR) analysis of target genes.

Gene	Forward Primer	Reverse Primer
*human β-ACTIN*	5′-GGAGACAGGTAACAGTTTCGG-3′	5′-CCAGCGGGGTGTTGGAGTTC-3′
*human ALPL*	5′-CCGTGGCAACTCTATCTTTGG-3′	5’-GCCCTACAGGATGGCAGTGA-3’
*human CCNB1*	5’-TGGATGCAGAAGATGGAGCTG-3’	5’-ACTGCTTGCTCTTCCTCAAGT-3’
*human COL1A1*	5’-GTGCGATGACGTGATCTGTG-3’	5’-GTGGGTGACTCTGAGCCG-3’
*human ID1*	5’-TTCAGCCAGTCGCCAAGAAT-3’	5’-TTTCCAGGCTCCTTAGGCAC-3’
*human MAP2*	5’-GCGGGTGCATCCAGTTTCT-3′	5′-CCTTGCAGACACCTCCTCTG-3′
*human MYF6*	5′-TGATAACGGCTAAGGAAGGAGG-3′	5′-CCACGATGGAAGAAAGGCAT-3′
*human MYOD1*	5′-TCCGACGGCATGATGGACTA-3′	5′-GGGCGCCTCGTTGTAGTAG-3′
*human MYOG*	5′-CGAATGCAGCTCTCACAGCG-3′	5′-CCGTGAGCAGATGATCCCC-3′
*human NEUROD1*	5′-CCCTTCCTTTGATGGACCCC-3′	5′-AAATGGTGAAACTGGCGTGC-3′
*human RUNX2*	5′-GAACCCAGAAGGCACAGACA-3′	5′-GGATGAGGAATGCGCCCTAA-3′
*human THY1*	5′-GGACTGCCGCCATGAGAATA-3′	5′-TGCTTCTTTGTCTCACGGGT-3′
*human TUBB3*	5′-GAGGCACCTCAGACACTCAC-3′	5′-CAGGCAGTCGCAGTTTTCAC-3′

## Data Availability

No new datasets were generated or deposited in publicly accessible repositories. The data supporting the findings of this study are available from the corresponding author upon reasonable request.

## Supplementary Materials

The following supporting information can be downloaded at: https://www.mdpi.com/article/10.3390/jfb17070336/s1, Figure S1: Preliminary qualitative comparison of CMSCs morphology on uncoated and gelatin-coated PETG substrates; Figure S2: Characterization of PETG-FDM substrates and cell viability assessment; Figure S3: Relative expression of osteogenic markers before and during osteogenic differentiation of CMSCs.
