# Supplementary material for: Polyethylene Terephthalate Glycol-Modified (PETG) as a Reusable and Biocompatible Substrate for Cell Culture Applications
Source: J Funct Biomater. 2026 Jul 11;17(7):336. doi: 10.3390/jfb17070336 (PMC13413215; doi:10.3390/jfb17070336)
Supplement: Supplementary file 1 [file jfb-17-00336-s001.zip › jfb-4373020-supplementary.pdf]

Supplementary Information (SI)

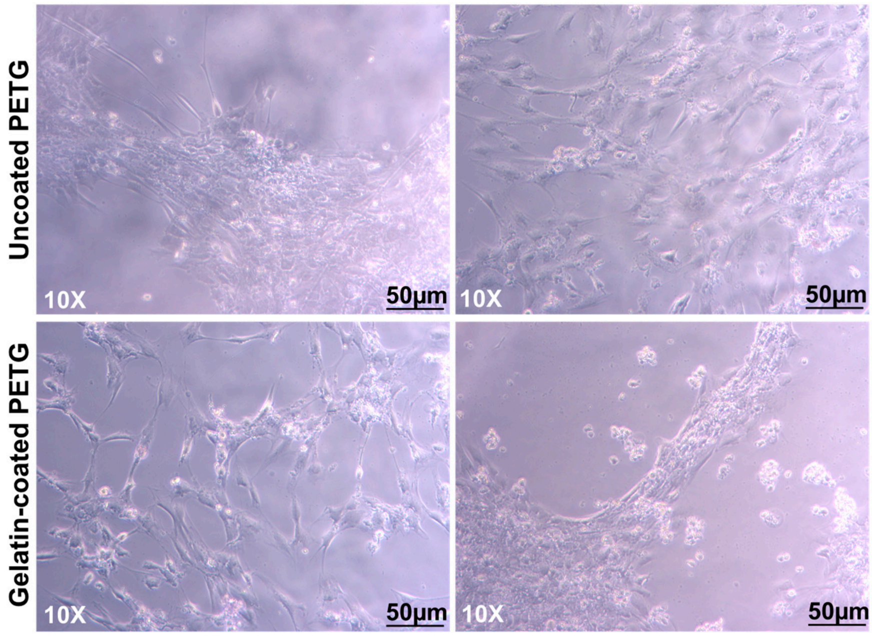

Figure S1. Preliminary qualitative comparison of CMSCs morphology on uncoated and gelatin-coated PETG substrates. Representative light microscopy images of CMSCs cultured for 24 h under proliferative conditions on uncoated PETG substrates (top row) and gelatin-coated PETG substrates (bottom row). Two representative fields are shown for each condition. CMSCs adhered under both conditions; however, gelatin-coated PETG showed a more homogeneous cell distribution and more reproducible cell morphology across the substrate surface. Scale bars: 50 µm.

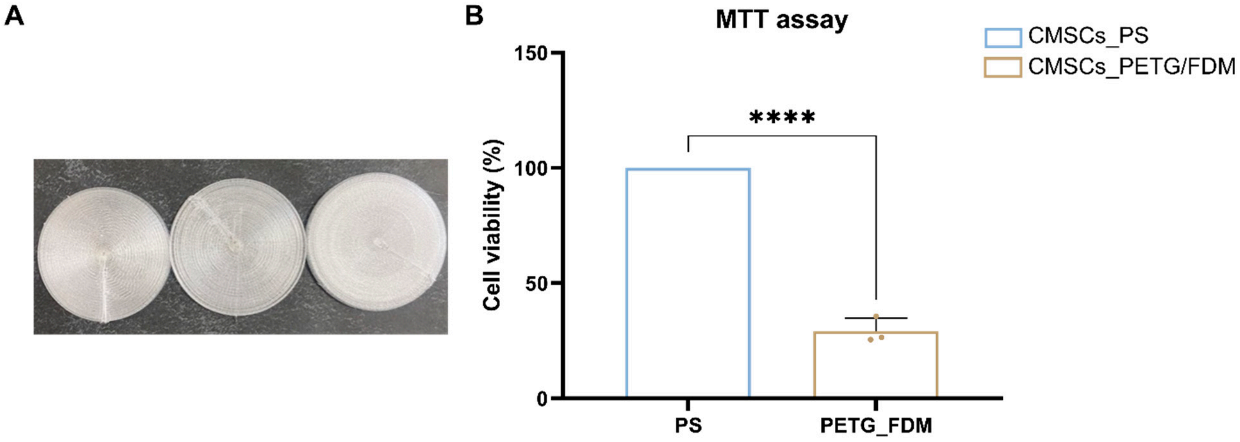

Figure S2. Characterization of PETG-FDM substrates and cell viability assessment. **A)** Representative images of PETG substrates fabricated via fused deposition modeling (FDM), highlighting the layered structure and surface morphology of the printed substrates; **B)** MTT assay results showing cell viability on PETG-FDM substrates after 48 hours of culture. Data are presented as mean (n = 3) ± SD, and statistical significance was assessed using an unpaired Student's t-test; $p \leq 0.0001$ *(****)*.

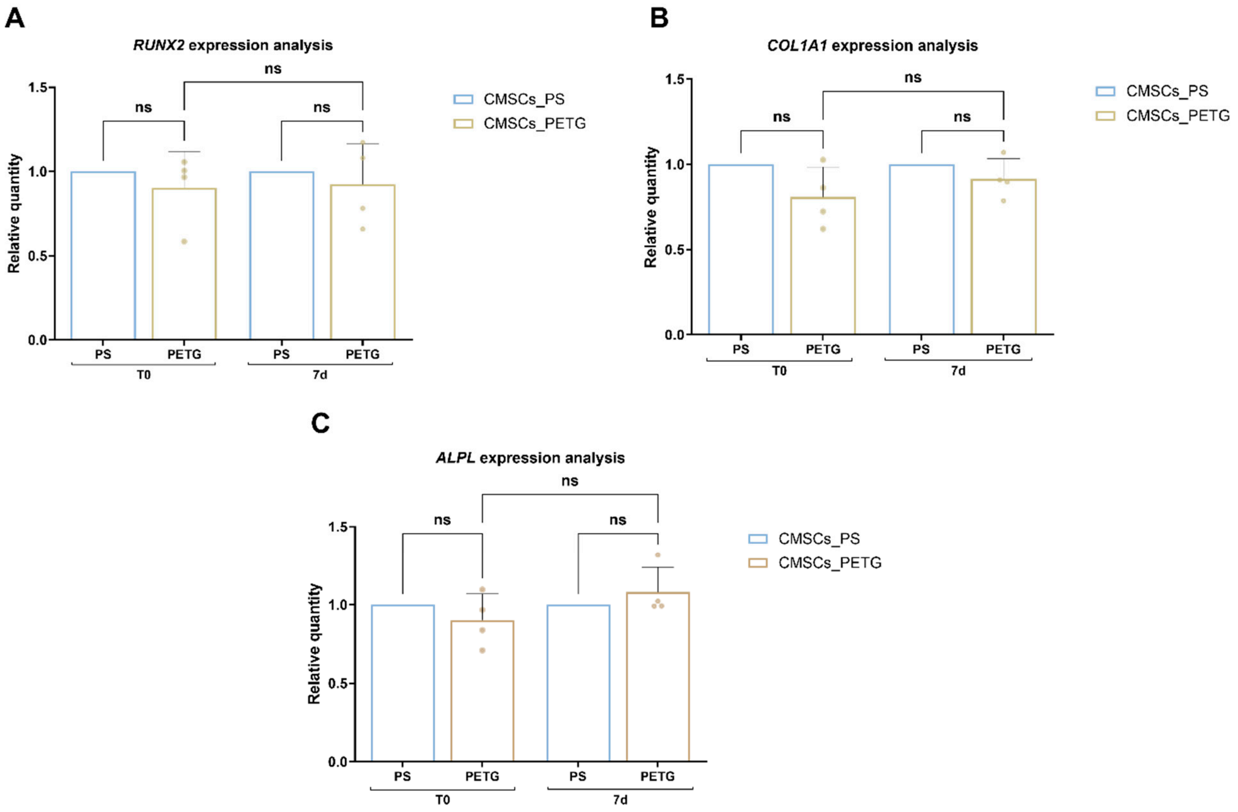

Figure S3. Relative expression of osteogenic markers before and during osteogenic differentiation of CMSCs. CMSCs were cultured on PETG and PS with proliferation medium for 72h (T0) and then in osteogenic medium for 7 days (7d). Relative transcript levels of key osteogenic markers such as *RUNX2* (**A**), *COL1A1* (**B**) and *ALPL* (**C**) were analyzed to compare gene expression between PETG and PS at each time point. Data are presented as mean (n=4) ± SD, and statistical significance was determined using one-way ANOVA; ns: not significant.
